# Diabetes, glycaemic traits and cardiovascular disease in females and males: Observational and Mendelian randomisation analyses in the UK Biobank

**DOI:** 10.1111/dom.16406

**Published:** 2025-04-21

**Authors:** Sophie C. de Ruiter, Lena Tschiderer, Diederick E. Grobbee, Ynte M. Ruigrok, Peter Willeit, Hester M. den Ruijter, A. Floriaan Schmidt, Sanne A. E. Peters

**Affiliations:** ^1^ Julius Center for Health Sciences and Primary Care University Medical Center Utrecht, Utrecht University Utrecht the Netherlands; ^2^ Institute of Clinical Epidemiology, Public Health, Health Economics, Medical Statistics and Informatics Medical University of Innsbruck Innsbruck Austria; ^3^ UMC Utrecht Brain Center, Department of Neurology and Neurosurgery Utrecht University, University Medical Center Utrecht Utrecht the Netherlands; ^4^ Ignaz Semmelweis Institute, Interuniversity Institute for Infection Research Medical University of Vienna Vienna Austria; ^5^ Department of Public Health and Primary Care University of Cambridge Cambridge UK; ^6^ Laboratory of Experimental Cardiology, Department of Cardiology University Medical Center Utrecht, University Utrecht Utrecht the Netherlands; ^7^ Department of Cardiology, Amsterdam Cardiovascular Sciences Amsterdam University Medical Centres, University of Amsterdam Amsterdam the Netherlands; ^8^ Institute of Cardiovascular Science Faculty of Population Health, University College London London UK; ^9^ Division Heart and Lungs, Department of Cardiology University Medical Center Utrecht, Utrecht University Utrecht the Netherlands; ^10^ UCL British Heart Foundation Research Accelerator London UK; ^11^ The George Institute for Global Health School of Public Health, Imperial College London London UK

**Keywords:** cardiovascular disease, diabetes, glycaemic traits, Mendelian randomisation, risk factors, sex differences

## Abstract

**Introduction:**

Observational studies have shown that the association between type 2 diabetes and cardiovascular disease (CVD) is stronger in females than in males. It remains unclear whether the causal effects of diabetes and glycaemic traits on CVD are also different between females and males.

**Methods:**

We performed sex‐stratified observational and Mendelian randomisation (MR) analyses in the UK Biobank to investigate the sex‐specific associations of type 2 diabetes and HbA1c with CVD outcomes (combined CVD, coronary heart disease [CHD], myocardial infarction, stroke, ischaemic stroke, intracerebral haemorrhage and subarachnoid haemorrhage). As secondary analyses, we performed sex‐stratified MR for the association of genetically proxied fasting glucose and insulin with CVD outcomes.

**Results:**

In observational analysis, diabetes was associated with a greater excess risk for CHD in females than in males (female‐to‐male ratio of hazard ratios 1.11 [95% CI 1.03, 1.21]). The association of HbA1c with CVD outcomes was similar in both sexes. In MR, the relationship between genetic liability to diabetes and CHD was similar in females and males (female‐to‐male ratio of odds ratios 0.98 [95% CI 0.91, 1.05]). No sex differences were found for the association between diabetes and stroke in both observational and MR analyses. Moreover, MR results on HbA1c, fasting glucose and fasting insulin were similar for females and males.

**Conclusion:**

This study suggests that causal effects of diabetes and glycaemic traits on CVD are similar in females and males.

## 1 INTRODUCTION

Type 2 diabetes is a major risk factor for cardiovascular disease (CVD), a leading cause of mortality worldwide, with high fasting glucose being the third most important metabolic risk factor for CVD mortality in both females and males.^1^ Previous studies have shown that the association between diabetes and several CVD outcomes is stronger in females than in males.^2^, ^3^, ^4^ However, since these studies were observational, potential biases such as residual or unmeasured confounding cannot be excluded. Consequently, it remains unclear whether the observed sex differences also translate into sex differences in causal effects.

Mendelian randomisation (MR) is a method to study potential causal effects of exposures (e.g., diabetes) on outcomes (e.g., CVD) by using genetic variants associated with the exposure of interest.^5^, ^6^ Previous sex‐combined MR studies have shown a causal relationship between diabetes and various cardiovascular outcomes.^7^, ^8^, ^9^ A previous sex‐specific MR on diabetes as a risk factor for CHD found a similar causal effect in females and males, which is different from observational evidence.^10^ Sex‐specific MR on the effects of glycaemic traits, such as HbA1c, fasting glucose and insulin, as well as a broader range of CVD outcomes (e.g., including not only CHD but also myocardial infarction, stroke, ischaemic stroke, intracerebral haemorrhage (ICH) and subarachnoid haemorrhage (SAH)), could provide further insight in the potentially sex‐specific mechanisms of diabetes as a risk factor for CVD.

In this study, we performed sex‐stratified observational and MR analyses to evaluate the sex‐specific associations of diabetes and glycaemic traits with the risk of different CVD outcomes, which were accompanied by MR analyses on the effect of fasting glucose and insulin.

## 2 METHODS

The results of this study are presented in accordance with the STROBE and STROBE‐MR guidelines. The STROBE and STROBE‐MR checklists are available in Tables S1 and S2.

### 2.1 Data source

For the observational analyses, data from the UK Biobank (UKB) were used, a prospective cohort study in the UK including over 500 000 participants aged 40–69 years, who were recruited between 2006 and 2010.^11^, ^12^ Participants attended one of the 22 centres across the UK for detailed baseline assessment that involved collection of extensive questionnaire data, physical measurements and biological samples. The UKB was approved by the North West Multi‐Centre Research Ethics Committee, and all participants provided written informed consent.

For the MR analyses, we applied a two‐sample MR design, using summary‐level GWAS data from European populations in the DIAGRAM and MAGIC consortia^13^, ^14^, ^15^ for genetic associations with the exposures and data from the UKB for genetic associations with CVD outcomes (more details are provided in the Supplementary Methods).

### 2.2 Study participants

For observational analyses, participants were excluded when they had a history of CVD (defined as CHD or stroke) at baseline according to data derived through linkage with routinely collected data from general practitioners, hospital admission and self‐reported data (from the first occurrence of disease data set released by the UKB^16^).

For MR analyses, we only included UKB participants with available high‐quality genetic data. Participants were excluded when they had a missing genotyping rate of more than 2%, were related, were outliers based on heterozygosity and missing rates or had a mismatch between genetic and reported sex. To minimise population stratification, we further excluded participants that were not self‐identified as White British and/or did not have a genetically validated British White ancestry based on the principal component analysis of the genotypes.

### 2.3 Definition of diabetes and glycaemic traits

Diabetes was defined as type 2 diabetes mellitus at baseline by combining self‐reported diagnosis and data derived through linkage with routinely collected data from general practitioners, hospital admissions and death records. Blood HbA1c levels were measured in mmol/mol with high‐performance liquid chromatography using the Bio‐Rad VARIANT II TURBO HbA1c analyser. We converted HbA1c levels from mmol/mol to percentage using the equation (0.09148 × HbA1c in mmol/mol) + 2.152.^17^ Fasting glucose and insulin levels were not measured in UKB participants.

Genetic liability to diabetes and genetically proxied levels of HbA1c, fasting glucose and fasting insulin were defined by selecting variants related to these traits from large‐scale meta‐analysis GWAS of the DIAGRAM and MAGIC consortia^13^, ^14^, ^15^ (Supplemental Methods). For diabetes, fasting glucose and insulin, sex‐specific GWAS data were available, which we used to define separate genetic instruments for females and males.

### 2.4 Genetic data and variant selection

We obtained individual‐level imputed data on genetic variants from the UKB. Participants were genotyped with the Affymetrix UK BiLEVE Axiom array and the Affymetrix UKB Axiom Array.^12^, ^18^ Genotype imputation was performed using the Haplotype Reference Consortium and the UK10K haplotype reference panel for the UKB.^19^

For each exposure (and in the case of diabetes, fasting glucose and insulin, for each sex), we selected variants (SNPs) based on a strong association with the exposure as reported by the corresponding GWAS using a *p*‐value threshold of 5 × 10^−8^, and on a low pairwise linkage disequilibrium (*r* ^2^ < 0.001) based on the 1000Genome reference panel, which includes only variants with minor allele frequencies higher than 0.01.^20^, ^21^ We checked whether genetic variants were reported on the same allele and harmonised the data accordingly. Variants were excluded if the INFO score was 0.9 or lower and when the variant was not in Hardy–Weinberg equilibrium using a *p*‐value threshold of 1 × 10^−5^.^22^ An overview of all selected variants per exposure is provided in Table S3.

### 2.5 Definition of endpoints

Cardiovascular outcomes were defined using clinical coding systems from linked health data, including International Classification of Diseases (ICD) codes for hospital admission data and death records, and Read codes (v2 and CTV3) for general practitioners' data, based on the first occurrence of disease framework released by the UKB, which provides mapping between the different coding systems and ICD‐10 codes.^21^ CVD was defined using ICD‐10 codes I20–I25, I60, I61, I63 and I64. We conducted separate analyses on major CVD outcomes including coronary heart disease (CHD) (I20–I25), acute myocardial infarction (MI) (I21), stroke (I60, I61, I63, I64), ischaemic stroke (I63, I64), ICH (I61) and SAH (I60).

For MR analyses, all events were considered, because they can be treated as incident since they occur after conception. For observational analyses, we only considered incident events after baseline, and follow‐up ended at the onset of the first CVD event, death, loss to follow‐up, or 31 December 2022, whichever occurred first.

### 2.6 Statistical analysis

The primary analysis involved both observational and MR analyses to study the associations between (genetic liability to) diabetes and (genetically proxied) HbA1c levels with CVD outcomes. Secondary analyses were conducted to study the associations between genetically proxied fasting glucose and insulin levels with CVD outcomes, using MR analysis exclusively.

#### 2.6.1 Observational analysis

Sex‐specific hazard ratios (HRs) with corresponding 95% confidence intervals (CIs) for the associations between diabetes and HbA1c with CVD were estimated by performing Cox regression analysis using age as the underlying time scale. We adjusted for sex, the Townsend deprivation index (an area‐based measure of socioeconomic status), systolic blood pressure, total cholesterol levels, smoking status, body mass index, use of lipid‐lowering medication and use of antihypertensives (Supplemental Methods). In the analyses of HbA1c as exposure, we also adjusted for diabetes status. We included an interaction term between each of these adjustment variables and sex. We also added a sex interaction term with each of the exposures to obtain female‐to‐male ratios of HRs (RHRs) and corresponding 95% CIs.

Missing values were imputed separately for females and males using multiple imputation by chained equations. This was carried out with 20 imputed data sets and 30 iterations for each sex (Supplemental Methods).

To ensure that differences between observational and MR results were not affected by potential variations between the population, we conducted a sensitivity analysis in the observational data in the same population as was selected for the MR analyses. For HbA1c, we also performed an observational analysis in a population restricted to non‐diabetics, to study the association in a population that is less likely to be treated for their elevated HbA1c.

#### 2.6.2 Mendelian randomisation analyses

Genetic associations of selected variants with CVD outcomes were estimated using logistic regression in the UKB data, for females and males separately. We adjusted for age at baseline and the first 16 genetic principal components.^23^

In our primary MR analysis, we performed inverse‐variance weighting. Sex‐specific models were used to obtain female‐to‐male ratios of ORs (RORs) and corresponding 95% CIs. We used the Cochran's *Q* test to assess the heterogeneity between estimates obtained using different variants and MR‐Egger to investigate potential horizontal pleiotropy (i.e., when the Egger intercept deviates from zero). Due to conservativeness under a low number of variants, results from these tests were not reported for analyses involving ten or fewer variants.^24^, ^25^ In addition, we performed simple median regression, weighted median regression and MR Pleiotropy Residual Sum and Outlier (MR‐PRESSO) analyses to assess the robustness of MR results to distinct assumptions about horizontal pleiotropy.^26^ For analysis involving more than four variants, we used the MR‐PRESSO distortion test to test for differences in estimates before and after excluding outlier variants that were detected by MR‐PRESSO.

As another sensitivity analysis, we repeated MR analysis for HbA1c by (1) adjusting for phenotypic diabetes and (2) restricting the population to non‐diabetics to account for the effect of diabetes treatment on HbA1c levels.

To assess instrumental variable strength, we report the *F*‐statistics (Supplemental Methods).^27^ The number of selected variants for each exposure and per estimate with corresponding *F*‐statistics are provided in Table S4.

Statistical analyses were conducted using R version 4.0.5 (R Foundation, Vienna, Austria). MR analyses were conducted using the R‐packages *MendelianRandomization* ^28^ and *MR‐PRESSO*.^26^ All tests were two‐sided, and *p*‐values of 0.05 or less were considered statistically significant.

## 3 RESULTS

Of 502 359 UKB participants eligible for inclusion, 468 838 participants had no history of CVD and were included in observational analyses. The mean age at recruitment was 56 (standard deviation [SD] 8) years and 56% were females (Table 1). At baseline, females were less likely to have diabetes (4% in females vs. 6% in males). The average age of diabetes diagnosis was similar in both sexes (54.4 years, SD 8.2 in females vs. 54.9 years, SD 7.8 in males), as well as the average level of HbA1c (35.7 mmol/mol, SD 5.8 in females vs. 36.1 mmol/L, SD 7.1 in males).

**TABLE 1:** Characteristics of study population in observational analysis.

Characteristics	No. of non‐missing values in females	Females (*n* = 261 920)	No. of non‐missing values in males	Males (*n* = 206 918)
Age, years	261 920	56.1 (8.0)	206 918	56.2 (8.2)
Ethnicity	260 714		205 570	
White		246 775 (94.2)		194 382 (93.9)
Other^a^		13 939 (5.3)		11 188 (5.4)
Systolic blood pressure, mm Hg	246 245	135.1 (19.2)	194 738	141.0 (17.4)
Total cholesterol, mmol/L	244 056	5.9 (1.1)	194 245	5.6 (1.1)
HbA1c, mmol/mol	242 422	35.7 (5.8)	192 958	36.1 (7.1)
Smoking status	260 514		205 697	
Never smoker		156 428 (59.7)		103 982 (50.3)
Former smoker		81 109 (31.0)		76 082 (36.8)
Current smoker		22 977 (8.8)		25 633 (12.4)
Body mass index, kg/m^2^	260 586	27.0 (5.1)	205 540	27.7 (4.2)
Socioeconomic status	261 606		206 649	
Townsend deprivation index score		−2.17 [−3.6, 0.4]		−2.17 [−3.7, 0.5]
Townsend deprivation thirds:				
Low (≥1.40)		49 097 (18.7)		40 676 (19.7)
Middle (≥ − 2.08 to <1.40)		78 601 (30.0)		60 374 (29.2)
High (<−2.08)		133 908 (51.1)		105 599 (51.0)
Type 2 diabetes	260 857	8916 (3.4)	205 784	11 982 (5.8)
Age at diagnosis of type 2 diabetes, years	3775	54.4 (8.2)	5350	54.9 (7.8)
Drug use				
Antihypertensive drugs	258 132	41 576 (15.9)	202 835	41 087 (19.9)
Lipid‐lowering drugs	258 132	27 208 (10.4)	202 835	34 458 (16.7)
Outcomes				
Cardiovascular disease		19 076 (7.3)		28 768 (13.9)
Coronary heart disease		14 450 (5.5)		23 458 (11.3)
Myocardial infarction		3192 (1.2)		6820 (3.3)
Stroke		4626 (1.8)		5310 (2.6)
Ischaemic stroke		3392 (1.3)		4388 (2.1)
Intracerebral haemorrhage		709 (0.3)		664 (0.3)
Subarachnoid haemorrhage		525 (0.2)		258 (0.1)

*Note*: Numbers are presented as mean (standard deviation), median [25th, 75th percentile], or number (percentage). Baseline characteristics are reported for the full study population in the observational analysis, stratified by sex.

^a^ Includes Asian or Asian British, Indian, Pakistani, Bangladeshi, any other Asian background, Chinese, black or black British, Caribbean, African, any other black background, other ethnic group, white and black Caribbean, white and black African, white and Asian and any other mixed background.

In the population included in the observational analysis, over a median follow‐up of 13.7 years (25th–75th percentile, 12.8–14.5), 19 076 (7.3%) females and 28 768 (13.9%) males experienced the combined CVD endpoint, including 14 450 (5.5%) females and 23 458 (11.3%) males with CHD (of which 3192 [1.2%] females and 6820 [3.3%] males with MI) and 4626 (1.8%) females and 5310 (2.6%) males with stroke (of which 3392 [1.3%] females with ischaemic stroke, 4388 [2.1%] males with ischaemic stroke, 709 [0.3%] females with ICH, 664 [0.3%] males with ICH, 525 [0.2%] females with SAH and 258 [0.1%] males with SAH). Characteristics of the population included in the observational analysis restricted to non‐diabetics are presented in Table S5.

Of all UKB participants eligible for inclusion, 337 386 were included in MR analyses. Characteristics of this population are presented in Tables S6 and S7 (restricted to non‐diabetics). The strength of instrumental variables was assessed using *F*‐statistics, which ranged from 19.5 to 142.2 (Table S4).

### 3.1 Primary analysis

**FIGURE 1:**
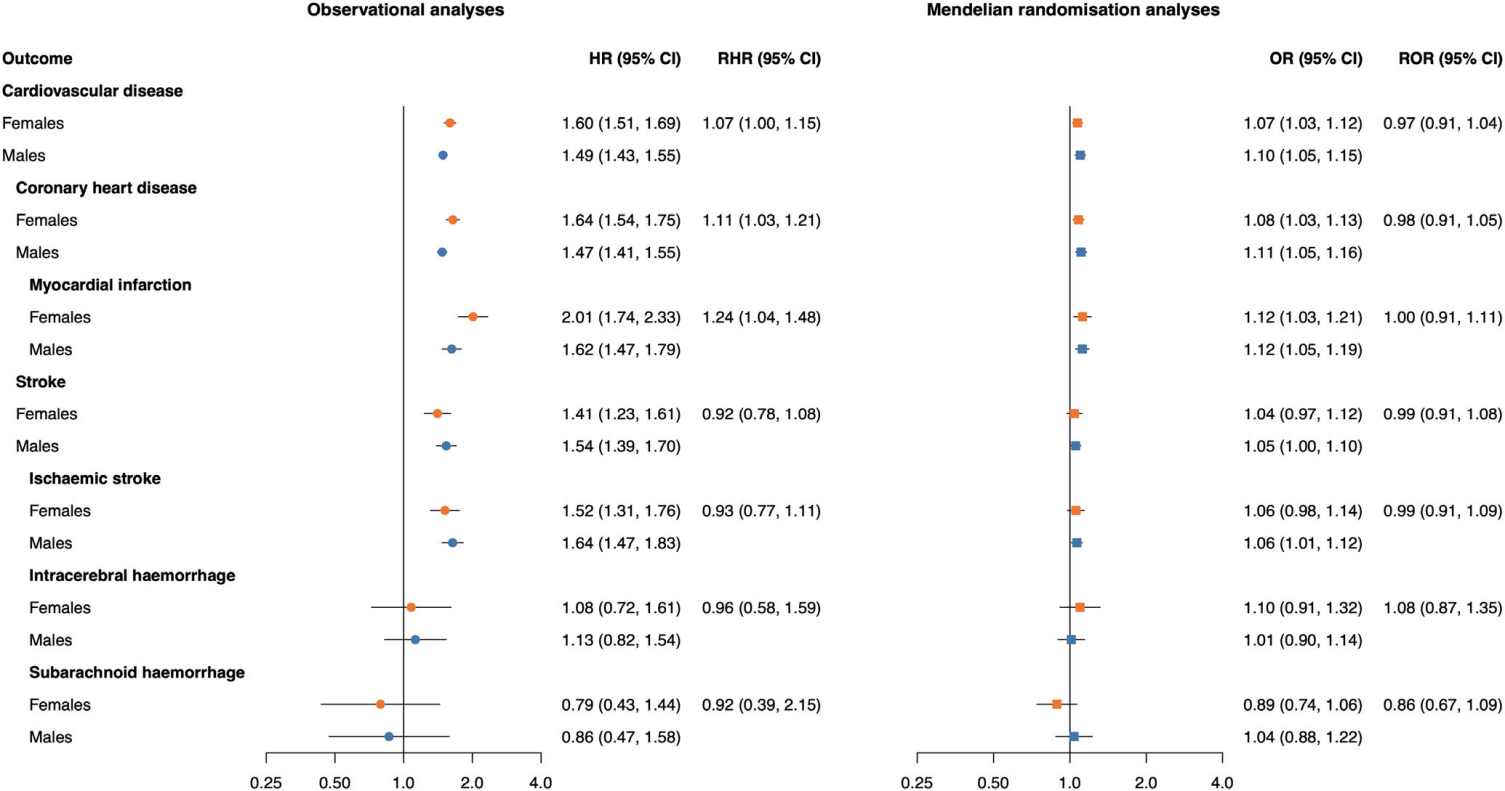
Cox regression estimates and Mendelian randomisation estimates of the association between type 2 diabetes and cardiovascular disease outcomes in females and males. MR estimates are from inverse‐variance weighted MR, and ORs can be interpreted as the effect per unit increase in log odds of genetic liability to diabetes. MR analyses were performed in 337 386 UK Biobank participants. Cox regressions were performed in 468 838 UK Biobank participants and were adjusted for sex, Townsend deprivation index (an area‐based measure of socioeconomic status), systolic blood pressure, total cholesterol levels, smoking status, body mass index, use of lipid‐lowering medication and use of antihypertensives, including an interaction term between each of these adjustment variables and sex. RHRs present the female‐to‐male ratios of HRs as obtained from an interaction term of diabetes with sex, and RORs present the female‐to‐male ratios of ORs as obtained from two separate MR analyses. CI, confidence interval; HR, hazard ratio; MR, Mendelian randomisation; OR, odds ratio; RHR, ratio of hazard ratios; ROR, ratio of odds ratios.

In observational analyses, diabetes was associated with a higher risk of CVD including CHD, MI, stroke and ischaemic stroke in both females and males (Figure 1). The observed association of diabetes was stronger in females than in males for CHD (RHR 1.11 [95% CI 1.03, 1.21]) and for MI (RHR 1.24 [95% CI 1.04, 1.48]). In MR analyses, genetic liability to diabetes was associated with an increased risk of CVD, CHD and MI in females and males with a similar effect in both sexes.

**FIGURE 2:**
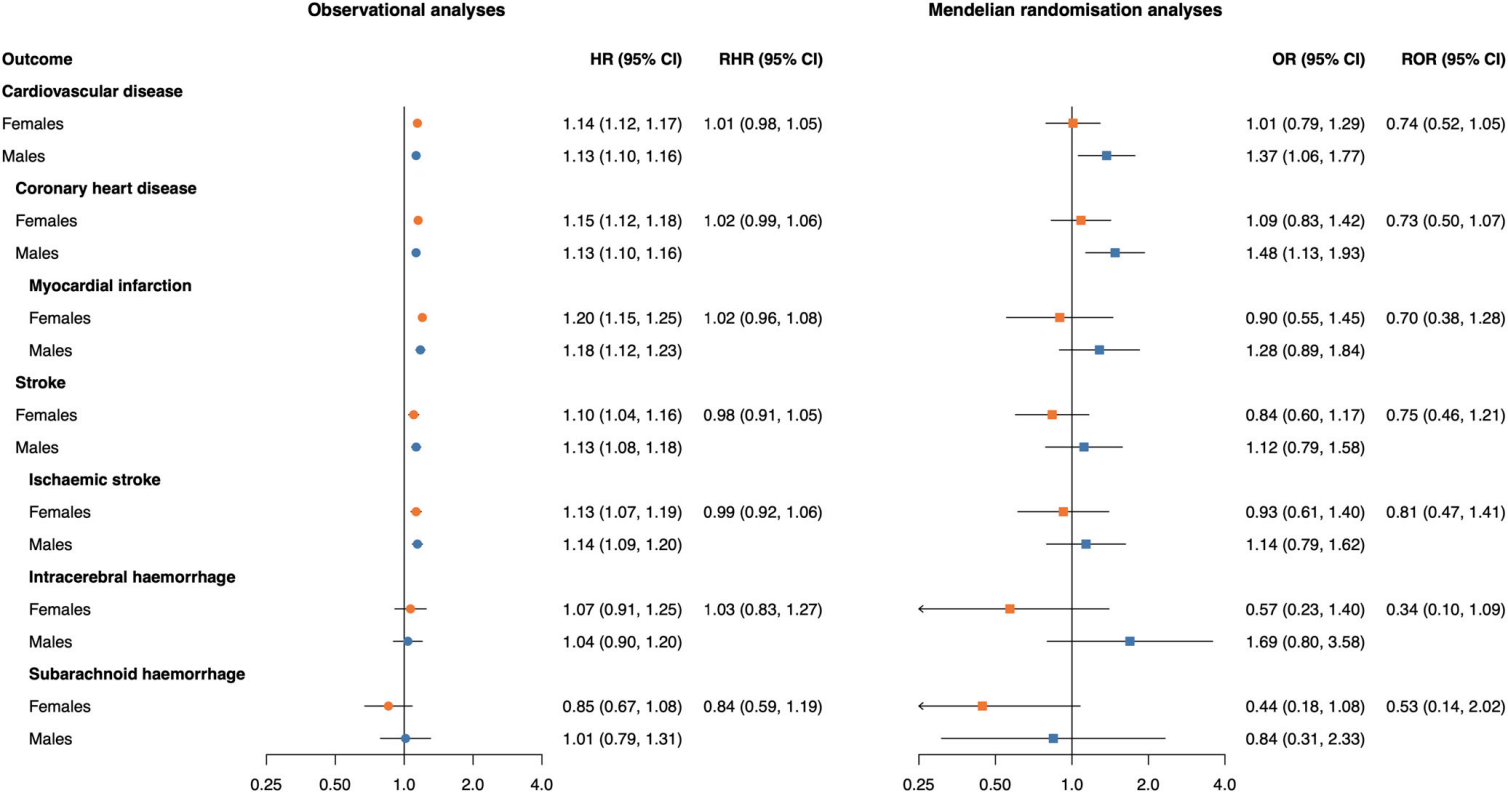
Cox regression estimates and Mendelian randomisation estimates of the association between HbA1c and cardiovascular disease outcomes in females and males. MR estimates are from inverse‐variance weighted MR and odds ratios (ORs) can be interpreted as the effect per 1% increase in genetically predicted HbA1c level. MR analyses were performed in 337 386 UK Biobank participants. Cox regressions were performed in 468 838 UK Biobank participants and adjusted for sex, type 2 diabetes status, Townsend deprivation index (an area‐based measure of socioeconomic status), systolic blood pressure, total cholesterol levels, smoking status, body mass index, use of lipid‐lowering medication and use of antihypertensives, including an interaction term between each of these adjustment variables and sex. RHRs present the female‐to‐male ratios of HRs as obtained from an interaction term of HbA1c and sex, and RORs present the female‐to‐male ratios of ORs as obtained from two separate MR analyses. CI, confidence interval; HR, hazard ratio; MR, Mendelian randomisation; OR, odds ratio; RHR, ratio of hazard ratios; ROR, ratio of odds ratios.

In observational analyses, higher levels of HbA1c were associated with an increased risk of CVD, CHD, MI, stroke and ischaemic stroke, with similar associations in females and males (Figure 2). In MR analyses, we found for males a relationship of genetically proxied HbA1c levels and CVD (OR 1.37 [95% CI 1.06, 1.77]) and CHD (OR 1.48 [95% CI 1.13, 1.93]), which was not found in females (OR 1.01 [95% CI 0.79, 1.29] for CVD, OR 1.09 [95% CI 0.83, 1.42] for CHD). No sex differences were found.

### 3.2 Secondary analysis

**FIGURE 3:**
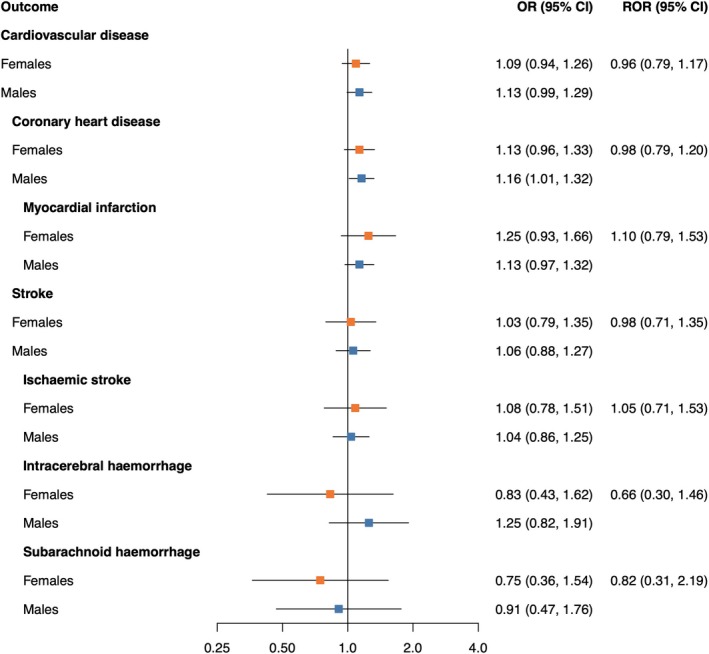
Mendelian randomisation estimates of the association between fasting glucose and cardiovascular disease outcomes in females and males. Estimates are from inverse‐variance weighted MR. The odds ratios (ORs) can be interpreted as the effect per 1 mmol/L increase in genetically predicted fasting glucose levels. MR analyses were performed in 337 386 UK Biobank participants. RORs present the female‐to‐male ratios of ORs as obtained from two separate MR analyses. CI, confidence interval; OR, odds ratio; ROR, ratio of odds ratios.

**FIGURE 4:**
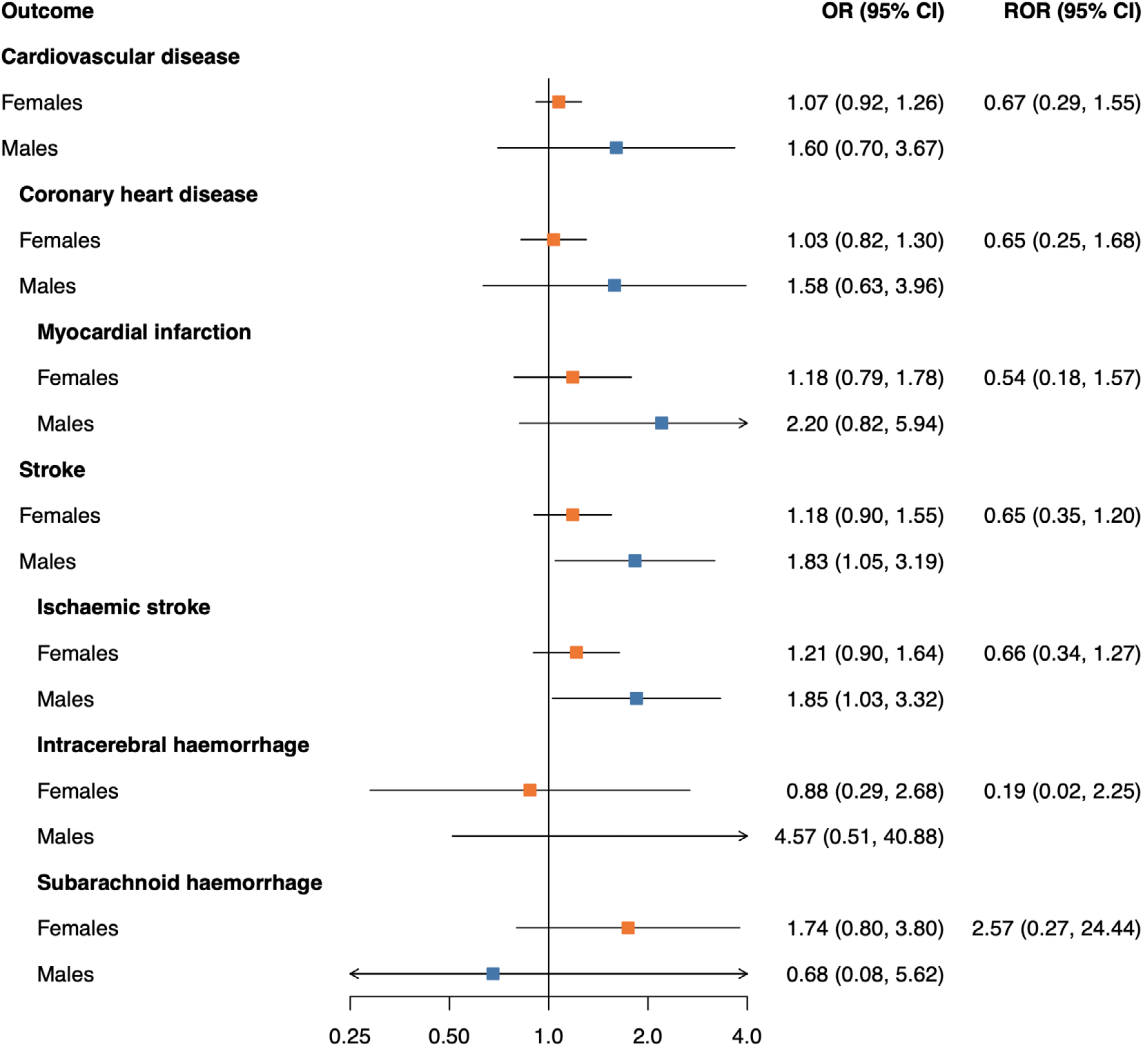
Mendelian randomisation estimates of the association between fasting insulin and cardiovascular disease outcomes in females and males. Estimates are from inverse‐variance weighted MR. The odds ratios (ORs) can be interpreted as the effect per one log unit increase in genetically predicted fasting insulin level. MR analyses were performed in 337 386 UK Biobank participants. RORs present the female‐to‐male ratios of ORs as obtained from two separate MR analyses. CI, confidence interval; OR, odds ratio; ROR, ratio of odds ratios.

Genetically proxied levels of fasting glucose (Figure 3) and fasting insulin (Figure 4) were not associated with CVD outcomes. No sex differences in the causal effects were found.

### 3.3 Sensitivity analyses

The results of observational analysis restricted to the same population as within the MR analyses (excluding those with a history of CVD at baseline) were directionally similar to the main analyses (Figures S1 and S2). The results of the MR analyses were broadly similar when applying simple and weighted median regression, except we found no significant relationship between genetically proxied HbA1c and CHD and MI in males using weighted median regression (Figures S3–S10). The *Q* test showed evidence of heterogeneity in the effect of diabetes and HbA1c SNPs on CVD outcomes for both females and males (Table S8). We further assessed pleiotropy using MR‐Egger regression, where we found no evidence for directional pleiotropy and MR‐PRESSO, which showed that outlier‐corrected results were directionally similar to the primary analysis (all distortion test *p*‐values >0.05, Table S9).

MR analyses for glycaemic traits conducted in the full population with adjustment for diabetes (Figures S11–S13), and observational and MR analyses for glycaemic traits in a non‐diabetic population (Figures S14–S16) provided similar results.

## 4 DISCUSSION

This large‐scale study used observational and MR analyses to assess associations of diabetes and glycaemic traits with CVD in females and males. While observational analyses showed a greater excess risk of CHD conferred by diabetes in females compared with males, no such difference was observed for stroke. The relationship between genetic liability to diabetes and CHD was similar for the sexes. There were also no sex differences in the effects of diabetes on stroke, nor in the effects of glycaemic traits on any of the CVD outcomes.

Previous observational studies have shown that the association between diabetes and CHD is stronger in females than in males. A meta‐analysis on 64 cohorts including 858 507 participants showed that the multiple‐adjusted relative risk for incident CHD associated with diabetes was 44% greater in females than in males (female‐to‐male ratio of relative risks 1.44 [95% CI 1.27, 1.63]).^3^ According to our MR analyses, the causal effect of diabetes on CHD is not likely to be different in females and males. The narrow confidence interval for the estimated ROR suggests that if a sex difference exists, it is likely to be small. MR estimates for the effect of diabetes on CHD are in line with previous evidence^10^ showing a similar causal effect based on MR in the UKB (female OR 1.13 [95% CI 1.08, 1.18], male OR 1.21 [95% CI 1.17, 1.26]). In contrast to the present MR, the previous study^10^ selected their instrumental variables from the sex‐combined DIAGRAM GWAS and weighted their instrumental variables by sex‐specific beta coefficients obtained from the DIAGRAM GWAS, while we used a fully sex‐specific approach to select the genetic variants for the instrumental variables. Sex differences have also been shown in the observational association between diabetes and stroke, with stronger associations in females than in males. A previous meta‐analysis on 64 cohorts including 775 385 participants found that compared to males, females with diabetes had a 27% higher increased risk of stroke associated with diabetes (female‐to‐male ratio of relative risks 1.27 [95% CI 1.10, 1.46]).^2^ In this study, we did not find sex differences in the observational analyses and also found no evidence for differences in the causal effects of diabetes on the risk of stroke between the sexes. The differences in results of our observational analyses as compared to previous studies may be explained by methodological differences. The previous meta‐analysis included cohorts with differing definitions of stroke and diabetes, which could have affected the findings. Another study in the UKB^29^ reported results similar to the meta‐analysis but defined outcomes without the use of self‐report or general practitioners' data, had a shorter follow‐up and relied on self‐reported diabetes cases. Higher levels of HbA1c were, independent of diabetes, associated with a higher risk of CHD and stroke, with similar magnitudes in females and males, which is in agreement with previous studies.^30^, ^31^, ^32^ In our MR analyses, we did not find sex differences in the causal effects of HbA1c either.

Previous MR studies have shown causal relationships of glycaemic trait levels with CVD risk. For example, fasting glucose has been shown to increase CVD risk in a sex‐combined MR analysis.^9^ In our sex‐stratified MR analyses, sex‐specific causal effect estimates may be different from the sex‐combined effects, and the reduced statistical power may have limited our ability to detect causal effects in some analyses.

Sex differences in the association between diabetes and CHD and stroke in observational studies may be partially explained by sex‐specific unmeasured or residual confounding.^33^, ^34^ Unequal distribution of confounding factors between females and males can lead to a bias in the estimated risk factor association that is specific to or stronger in one of the sexes. While we adjusted for several potential confounders and included interaction terms with sex, other factors might still be involved. Also, to ensure identical models in females and males, we did not account for female‐specific risk factors such as gestational diabetes, which increases the risk of developing diabetes and CHD.

Sex differences in body anthropometry and patterns of adipose tissue storage may also contribute to sex differences in the observational analyses in our study. Previous studies have shown that, at the time of diabetes diagnosis, females have a worse cardiometabolic profile (across, for example, glycaemic traits such as HbA1c and fasting glucose) and higher BMI as compared to males.^35^ Females are more likely to store fat subcutaneously, while males are more likely to have more visceral fat,^36^ which leads to a faster insulin resistance and diabetes development in males. Consequently, females are often in a prediabetic state for a longer period than males prior to diabetes diagnosis,^37^ leading to a longer exposure to the risk‐increasing cardiometabolic state in females. These factors could contribute to an increased risk associated with diabetes in females compared to males.

Alternatively, sex differences in risk factor management may contribute to our findings. In general, females are perceived to have a lower cardiovascular risk than males, and as a consequence, control of diabetes risk factors may be worse in females, increasing their risk of CHD.^38^ However, sex differences in risk factor management are not specific to people with diabetes^35^ and our MR results indicate that the prevention and treatment of diabetes should be of equal priority in both sexes.

### 4.1 Strengths and limitations

Our study has several strengths. We sourced a large sample of UKB participants to study the associations of diabetes and glycaemic traits with several CVD outcomes in a sex‐stratified manner. We performed both observational and MR analyses. MR, however, relies on three assumptions. First, it is assumed that the selected genetic variants are strongly associated with the exposure of interest. This assumption is likely to hold when the instruments are selected from large‐scale meta‐analyses of GWAS data. Moreover, the *F*‐statistics we calculated indicate a low risk of weak instrument bias, and because we used two‐sample MR with non‐overlapping samples, any weak instrument bias would have biased our results towards the null, reducing power rather than increasing the risk of type 1 errors.^39^ The second assumption of MR is that there is no shared cause (i.e., a confounder) between the genetic variants and either the risk factor or the outcome. This assumption generally holds in MR, as an individual's genotype is determined at gametogenesis and is rarely affected by environmental exposures throughout life. A factor that is associated with both genetic variants and outcomes is population structure, for which we aimed to correct by restricting the exposure and outcome associations to the European population and by correcting for the first 16 principal components.^23^ In a MR sensitivity analysis for HbA1c, we accounted for the effect of diabetes treatment on HbA1c by either adjusting for phenotypic diabetes or restricting the analysis to non‐diabetics. However, this does introduce index event bias, a form of collider bias which induces an association between the genetic instrument and potential confounders, which can affect causal effect estimates. The third assumption is the absence of horizontal pleiotropy, meaning that instruments are only associated with the outcome through the risk factor. The between‐variant heterogeneity indicated by the *Q* test could be indicative of potential bias due to pleiotropy. However, estimates were broadly similar in the robust methods that make different assumptions with respect to pleiotropy. In addition, the *Q* test relies on the assumption that all valid instruments identify the same causal parameter and if this does not hold, it may over‐reject the null.^40^ Also, the *Q* test does not give information on the source of the heterogeneity, which could be pleiotropy or other reasons such as confounding by population stratification.^24^ Therefore, we further assessed pleiotropy using MR‐Egger regression, where we found no evidence for directional pleiotropy. As MR‐Egger results are sensitive to outlying SNPs,^41^ we further investigated the risk of directional pleiotropy using MR‐PRESSO, which focuses on outliers. This gave robust results.

In this study, we compared the ratio of HRs derived from observational analyses with the ratio of ORs from MR analyses. HRs provide a measure of time‐to‐event relationships and were adjusted for confounders, offering conditional estimates. In contrast, ORs from MR analyses reflect lifetime genetic liability to the exposure and are marginal estimates unaffected by confounding factors. Our focus on sex differences through the evaluation of sex interactions is expected to minimise the impact of these differences. In addition, it is possible that the associations of diabetes and HbA1c levels with CVD outcomes vary with age, potentially impacting the observed magnitude of sex‐specific trends. Moreover, as highlighted above and in a previous review,^35^ sex differences in the distribution of adipose tissue may contribute to sex differences in the association between diabetes and CVD. We adjusted our observational analyses for BMI. However, BMI may not fully capture specific distributions of adipose tissues. Future studies should, therefore, also take into account different patterns of storage of adipose tissue (e.g., obtained by Dual‐energy X‐ray Absorptiometry or bioelectrical impedance analysis).^35^ Another limitation in our study is the unavailability of sex‐specific GWAS for HbA1c, which required us to rely on sex‐combined GWAS. This might invalidate MR results in case of considerable heterogeneity of the genetic effects between the sexes. Although the high *F*‐statistics suggest that the instruments sufficiently proxied the HbA1c levels in both sexes, future studies using sex‐specific GWAS data on HbA1c would be valuable to validate our findings. In addition, all GWAS on glycaemic traits we used were conducted in non‐diabetics. While this reduces the risk of biased genetic effect estimates as glycaemic trait levels are less affected by treatment of diabetes, it also reduces power as compared to MR analysis on the effect of diabetes where the full population can be used. Additionally, our secondary analysis on fasting insulin had limited statistical power (especially for males). This may partly be explained because only a few SNPs were selected for the instrumental variables. Therefore, these findings should be interpreted with caution and further large‐scale sex‐specific GWASs are needed to potentially identify additional SNPs to genetically proxy fasting insulin. Similarly, the outcomes of ICH and SAH were limited and the resulting higher uncertainty of the underlying results needs to be considered when interpreting the results.

In this study, we focused on CHD and stroke. However, sex differences in the relationship between diabetes and other CVD outcomes have also been described. Previous observational studies reported a higher excess risk of heart failure^42^ and atrial fibrillation^43^ related to diabetes in women as compared to men, while no sex differences have been found in the relationship between diabetes and peripheral artery disease.^44^ Future studies are needed to assess the sex‐specific causal role of diabetes and glycaemic traits on other cardiovascular outcomes, including heart failure, atrial fibrillation and peripheral artery disease. A final limitation is the generalisability of our results to non‐European populations. The UKB predominantly includes individuals of European ancestry. In order to minimise the risk of bias due to population stratification, we also used GWAS data from European populations to genetically proxy the exposures of interest. However, a drawback of this approach is that our results may not be generalisable to non‐European populations. Consequently, future MR studies need to be performed in order to better understand the potential causal relationship between diabetes and glycaemic traits with CVD in other non‐European populations. Additionally, UKB participants are on average healthier and have a higher socioeconomic status than the general UK population.^45^ However, as described before,^45^ this should not be seen as a main limitation as the UKB was mainly designed to assess associations between exposure and disease and still includes participants from various population segments with different levels of socioeconomic statuses.

## 5 CONCLUSION

The sex differences in associations between diabetes and CHD were not likely to reflect a sex difference in the causal effects. This study suggests similar causal effects of diabetes and glycaemic traits on CVD in females and males.

## AUTHOR CONTRIBUTIONS

S.C. de Ruiter, L. Tschiderer and S.A.E. Peters designed the study. S.C. de Ruiter and L. Tschiderer accessed that data, verified the data and performed analyses. S.C. de Ruiter drafted the manuscript. All the authors provided critical input on the analysis, as well as the drafted manuscript.

## FUNDING INFORMATION

This research was funded in whole or in part by the Austrian Science Fund (FWF) [grant DOI: 10.55776/T1253]. For open access purposes, the author has applied a CC BY public copyright licence to any author‐accepted manuscript version arising from this submission. S.C. de Ruiter and S.A.E. Peters are supported by a VIDI Fellowship (project number 09150172010050) from the Dutch Organisation for Health Research and Development (ZonMW) awarded to S.A.E. Peters. Y.M. Ruigrok received funding from the Dutch Heart Foundation (Dekker grant 03‐001‐2022‐0157) and the European Research Council under the European Union's Horizon 2020 research and innovation program (grant agreement 852173). H.M. den Ruijter received funding by Dutch Cardiovascular Alliance 2020B004. A.F. Schmidt is supported by British Heart Foundation AA/18/6/34223 and PG/22/10989. A.F. Schmidt received additional support from the National Institute for Health Research University College London Hospitals Biomedical Research Centre, the Rosetrees Trust and by the UK Research and Innovation (UKRI) under the UK government's Horizon Europe funding guarantee EP/Z000211/1.

## CONFLICT OF INTEREST STATEMENT

P. Willeit reports consultancy fees from Novartis Pharmaceuticals unrelated to the present work.

## PEER REVIEW

The peer review history for this article is available at https://www.webofscience.com/api/gateway/wos/peer‐review/10.1111/dom.16406.

## ROLE OF FUNDERS

The study sponsor/funder was not involved in the design of the study; the collection, analysis and interpretation of data; writing the report; and did not impose any restrictions regarding the publication of the report.

## Supporting information


Data S1.

## Data Availability

The UK Biobank data used in this study is available upon request via the UKB website (https://www.ukbiobank.ac.uk/enable-your-research/apply-for-access). Genetic associations with fasting glucose and insulin and HbA1c levels have been published previously and can be downloaded from www.magicinvestigators.org.^14^, ^15^ Genetic associations with type 2 diabetes have been published previously and can be downloaded from https://diagram-consortium.org/downloads.html.^13^
