# Supplementary material for: Diabetes, glycaemic traits and cardiovascular disease in females and males: Observational and Mendelian randomisation analyses in the UK Biobank
Source: Diabetes Obes Metab. 2025 Apr 21;27(7):3789–99. doi: 10.1111/dom.16406 (PMC12146462; doi:10.1111/dom.16406)
Supplement: Supplementary file 1 — Data S1. [file DOM-27-3789-s001.zip › dom16406-sup-0001-supinfo/dom16406-sup-0001-supinfo.docx]

SUPPLEMENTAL MATERIAL

Supplemental Methods

**Genetic data**

For type 2 diabetes, we used a GWAS of individuals with type 2 diabetes (30,053 females and 41,846 males) and controls (434,336 females and 383,767 males) of European ancestry conducted by the DIAGRAM consortium (13). Sex-specific genetic associations (effect estimates and standard errors) were used. We used GWAS estimates obtained from analyses excluding the UK Biobank to avoid sample overlap.

For HbA1c, we used sex-combined GWAS (as no sex-specific GWAS was available) from the MAGIC consortium (14) which meta-analysed genetic associations from 42 GWAS in up to 146,806 non-diabetic adults of European Ancestry, excluding UKB participants.

For fasting glucose and fasting insulin, we used a sex-specific GWAS from the MAGIC consortium (15). Summary statistics were available from a meta-analysis on GWAS performed in up to 73,089 females and 67,506 males for the sex-specific associations with fasting glucose, and up to 50,404 females and 47,806 males for the sex-specific associations with fasting insulin. All meta-analyses were performed in adults of European descent without diabetes, excluding UKB participants. Our study used sex-specific beta coefficients and standard errors.

**Definition of additional variables**

Body mass index was calculated by dividing weight in kg by height in m^2^. Smoking status was self-reported and divided into never, former, and current tobacco smoking. Systolic and diastolic blood pressure were measured using automated reading and were averaged across two measurements taken a few moments apart. Total cholesterol, high-density lipoprotein cholesterol, low-density lipoprotein cholesterol, triglycerides, and high-sensitivity C-reactive protein were measured on a Beckman Coulter AU5800 platform (assay manufacturer Beckman Coulter [UK], Ltd). Lipoprotein(a) was measured on a Beckman Coulter AU5800 platform (assay manufacturer Randox Bioscience, UK). All adjustment variables were obtained at study baseline, i.e. at time of recruitment into the UKB.

**Multiple imputation of missing values**

Missing values were imputed using multiple imputation by chained equations with 20 data sets and 30 iterations. The imputation model included the following variables: age at baseline, smoking status, total cholesterol, high-density lipoprotein cholesterol, low-density lipoprotein cholesterol, triglycerides, Lipoprotein(a), HbA1c, body mass index, type 2 diabetes status, systolic blood pressure, diastolic blood pressure, Townsend index, C-reactive protein, eGFR, the use of lipid-lowering medication, the use of antihypertensives, and the Nelson-Aalen estimator for CVD. Predictive mean matching (pmm) was used for continuous variables, logistic regression (logreg) for binary variables and polytomous regression (polyreg) for categorical variables with multiple levels. If two predictors correlated strongly with each other (Pearson correlation coefficient higher than 0.7 or lower than -0.7), we selected the predictor with the greater correlation to the imputed variable for predicting that variable. Imputed results were pooled using Rubin’s rule.

**Instrumental variable strength**

To assess the strength of our instruments, we calculated the F-statistics for each exposure. We used a different approach for binary and continuous exposures, and for exposures that were and that were not available in the UKB.

For diabetes, we conducted logistic regression with diabetes status as dependent variable and all instruments as independent variables in the first imputed UKB data set. F-statistics were obtained from a comparison of the full model with a null model containing only an intercept. For HbA1c, we conducted linear regression with HbA1c status as dependent variable and all instruments as independent variables among non-diabetic individuals in the first imputed UKB data set to obtain the F-statistic.

For glucose and insulin (which were not available in the UKB), we obtained the F-statistic using the formula F = (R^2^ / k) × ((n-k-1) / (1-R^2^)) (where R^2^ was obtained from the MR Steiger directionality test, k is the number of SNPs in the instrumental variable and n is the total sample size) (27).

**Supplementary Tables**

**Table S1. STROBE checklist**

|  | Item No. | Recommendation | Page No. |
| --- | --- | --- | --- |
| Title and abstract | 1 | (a) Indicate the study’s design with a commonly used term in the title or the abstract | 1, 2 |
|  |  | (b) Provide in the abstract an informative and balanced summary of what was done and what was found | 2 |
| **Introduction** | | | |
| Background/rationale | 2 | Explain the scientific background and rationale for the investigation being reported | 4 |
| Objectives | 3 | State specific objectives, including any prespecified hypotheses | 4 |
| **Methods** | | | |
| Study design | 4 | Present key elements of study design early in the paper | 5 |
| Setting | 5 | Describe the setting, locations, and relevant dates, including periods of recruitment, exposure, follow-up, and data collection | 5 |
| Participants | 6 | (a) Give the eligibility criteria, and the sources and methods of selection of participants. Describe methods of follow-up | 5 |
|  |  | (b) For matched studies, give matching criteria and number of exposed and unexposed | NA |
| Variables | 7 | Clearly define all outcomes, exposures, predictors, potential confounders, and effect modifiers. Give diagnostic criteria, if applicable | 5-7 |
| Data sources/ measurement | 8* | For each variable of interest, give sources of data and details of methods of assessment (measurement). Describe comparability of assessment methods if there is more than one group | 5, 6 |
| Bias | 9 | Describe any efforts to address potential sources of bias | 7, 8, Supplemental Methods |
| Study size | 10 | Explain how the study size was arrived at | 5, 10 |
| Quantitative variables | 11 | Explain how quantitative variables were handled in the analyses. If applicable, describe which groupings were chosen and why | 6 |
| Statistical methods | 12 | (a) Describe all statistical methods, including those used to control for confounding | 7, 8, Supplemental Methods |
|  |  | (*b*) Describe any methods used to examine subgroups and interactions |  |
|  |  | (*c*) Explain how missing data were addressed |  |
|  |  | (*d*) If applicable, explain how loss to follow-up was addressed |  |
|  |  | (*e*) Describe any sensitivity analyses |  |
| **Results** |  |  |  |
| Participants | 13* | (a) Report numbers of individuals at each stage of study—eg numbers potentially eligible, examined for eligibility, confirmed eligible, included in the study, completing follow-up, and analysed | 10 |
|  |  | (b) Give reasons for non-participation at each stage |  |
|  |  | (c) Consider use of a flow diagram |  |
| Descriptive data | 14* | (a) Give characteristics of study participants (eg demographic, clinical, social) and information on exposures and potential confounders | 10, Table 1 |
|  |  | (b) Indicate number of participants with missing data for each variable of interest |  |
|  |  | (c) Summarise follow-up time (eg, average and total amount) |  |
| Outcome data | 15* | Report numbers of outcome events or summary measures over time | Table 1 |
| Main results | 16 | (*a*) Give unadjusted estimates and, if applicable, confounder-adjusted estimates and their precision (eg, 95% confidence interval). Make clear which confounders were adjusted for and why they were included | Figure 1 - 2 |
|  |  | (*b*) Report category boundaries when continuous variables were categorized |  |
|  |  | (*c*) If relevant, consider translating estimates of relative risk into absolute risk for a meaningful time period |  |
| Other analyses | 17 | Report other analyses done—eg analyses of subgroups and interactions, and sensitivity analyses | Figure S1, S2, S14 |
| **Discussion** |  |  |  |
| Key results | 18 | Summarise key results with reference to study objectives | 12 |
| Limitations | 19 | Discuss limitations of the study, taking into account sources of potential bias or imprecision. Discuss both direction and magnitude of any potential bias | 14-16 |
| Interpretation | 20 | Give a cautious overall interpretation of results considering objectives, limitations, multiplicity of analyses, results from similar studies, and other relevant evidence | 16 |
| Generalisability | 21 | Discuss the generalisability (external validity) of the study results | 16 |
| **Other information** |  |  |  |
| Funding | 22 | Give the source of funding and the role of the funders for the present study and, if applicable, for the original study on which the present article is based | 17 |
|  |  |  |  |

**Table S2. STROBE-MR checklist**

| Item No. | Section | Checklist item | Page No. |
| --- | --- | --- | --- |
| 1 | Title and abstract | Indicate Mendelian randomization (MR) as the study’s design in the title and/or the abstract if that is a main purpose of the study | 1-2 |
|  | **Introduction** |  |  |
| 2 | Background | Explain the scientific background and rationale for the reported study. What is the exposure? Is a potential causal relationship between exposure and outcome plausible? Justify why MR is a helpful method to address the study question | 4 |
| 3 | Objectives | State specific objectives clearly, including pre-specified causal hypotheses (if any). State that MR is a method that, under specific assumptions, intends to estimate causal effects | 4 |
|  | **Methods** |  |  |
| 4 | Study design and data sources | Present key elements of the study design early in the article. Consider including a table listing sources of data for all phases of the study. For each data source contributing to the analysis, describe the following: |  |
|  | a) | Setting: Describe the study design and the underlying population, if possible. Describe the setting, locations, and relevant dates, including periods of recruitment, exposure, follow-up, and data collection, when available. | 5-7, Supplementary Tables S6 and S7, Supplementary Methods |
|  | b) | Participants: Give the eligibility criteria, and the sources and methods of selection of participants. Report the sample size, and whether any power or sample size calculations were carried out prior to the main analysis | 5 |
|  | c) | Describe measurement, quality control and selection of genetic variants | 5-7 |
|  | d) | For each exposure, outcome, and other relevant variables, describe methods of assessment and diagnostic criteria for diseases | 6-7 |
|  | e) | Provide details of ethics committee approval and participant informed consent, if relevant | 5 |
| 5 | Assumptions | Explicitly state the three core IV assumptions for the main analysis (relevance, independence and exclusion restriction) as well assumptions for any additional or sensitivity analysis | 15 |
| 6 | Statistical methods: main analysis | Describe statistical methods and statistics used |  |
|  | a) | Describe how quantitative variables were handled in the analyses (i.e., scale, units, model) | Table 1 |
|  | b) | Describe how genetic variants were handled in the analyses and, if applicable, how their weights were selected | 6-7 |
|  | c) | Describe the MR estimator (e.g. two-stage least squares, Wald ratio) and related statistics. Detail the included covariates and, in case of two-sample MR, whether the same covariate set was used for adjustment in the two samples | 9 |
|  | d) | Explain how missing data were addressed | NA |
|  | **e)** | If applicable, indicate how multiple testing was addressed | NA |
| 7 | Assessment of assumptions | Describe any methods or prior knowledge used to assess the assumptions or justify their validity | 9 |
| 8 | Sensitivity analyses and additional analyses | Describe any sensitivity analyses or additional analyses performed (e.g. comparison of effect estimates from different approaches, independent replication, bias analytic techniques, validation of instruments, simulations) | 9 |
| 9 | Software and pre-registration |  |  |
|  | a) | Name statistical software and package(s), including version and settings used | 9-10 |
|  | b) | State whether the study protocol and details were pre-registered (as well as when and where) | NA |
|  | **Results** |  |  |
| 10 | Descriptive data |  |  |
|  | a) | Report the numbers of individuals at each stage of included studies and reasons for exclusion. Consider use of a flow diagram | 10 |
|  | b) | Report summary statistics for phenotypic exposure(s), outcome(s), and other relevant variables (e.g. means, SDs, proportions) | Supplementary Tables S6 and S7 |
|  | c) | If the data sources include meta-analyses of previous studies, provide the assessments of heterogeneity across these studies | Reference of source GWAS at page 6 |
|  | d) | For two-sample MR:  i.  Provide justification of the similarity of the genetic variant-exposure associations between the exposure and outcome samples  ii.  Provide information on the number of individuals who overlap between the exposure and outcome studies | Supplemental Methods, page 5, and for cohorts used in source GWAS, see reference page 6 |
| 11 | Main results |  |  |
|  | a) | Report the associations between genetic variant and exposure, and between genetic variant and outcome, preferably on an interpretable scale | Supplementary Table S3 and data availability for GWAS summary statistics |
|  | b) | Report MR estimates of the relationship between exposure and outcome, and the measures of uncertainty from the MR analysis, on an interpretable scale, such as odds ratio or relative risk per SD difference | Figure 1-4 |
|  | c) | If relevant, consider translating estimates of relative risk into absolute risk for a meaningful time period | NA |
|  | d) | Consider plots to visualize results (e.g. forest plot, scatterplot of associations between genetic variants and outcome versus between genetic variants and exposure) | Figure 1-4 |
| 12 | Assessment of assumptions |  |  |
|  | a) | Report the assessment of the validity of the assumptions | 15, Table S4, S8, S9, Supplementary Figures S3-10 |
|  | b) | Report any additional statistics (e.g., assessments of heterogeneity across genetic variants, such as *I^2^*, Q statistic or E-value) | Supplementary Table S8 |
| 13 | Sensitivity analyses and additional analyses |  |  |
|  | a) | Report any sensitivity analyses to assess the robustness of the main results to violations of the assumptions | 15, Table S4, S8, S9, Supplementary Figures S3-10 |
|  | b) | Report results from other sensitivity analyses or additional analyses | Figures 1-4, Supplementary Figures S1, S2, S11-16 |
|  | c) | Report any assessment of direction of causal relationship (e.g., bidirectional MR) | NA |
|  | d) | When relevant, report and compare with estimates from non-MR analyses | 11, Figure 1-4 |
|  | e) | Consider additional plots to visualize results (e.g., leave-one-out analyses) | Supplementary Figures S3-10 |
|  | **Discussion** |  |  |
| 14 | Key results | Summarize key results with reference to study objectives | 12 |
| 15 | Limitations | Discuss limitations of the study, taking into account the validity of the IV assumptions, other sources of potential bias, and imprecision. Discuss both direction and magnitude of any potential bias and any efforts to address them | 15, 16 |
| 16 | Interpretation |  |  |
|  | a) | Meaning: Give a cautious overall interpretation of results in the context of their limitations and in comparison with other studies | 12-14 |
|  | b) | Mechanism: Discuss underlying biological mechanisms that could drive a potential causal relationship between the investigated exposure and the outcome, and whether the gene-environment equivalence assumption is reasonable. Use causal language carefully, clarifying that IV estimates may provide causal effects only under certain assumptions | 13, 14 |
|  | c) | Clinical relevance: Discuss whether the results have clinical or public policy relevance, and to what extent they inform effect sizes of possible interventions | 14 |
| 17 | Generalizability | Discuss the generalizability of the study results (a) to other populations, (b) across other exposure periods/timings, and (c) across other levels of exposure | 16 |
|  | **Other information** |  |  |
| 18 | Funding | Describe sources of funding and the role of funders in the present study and, if applicable, sources of funding for the databases and original study or studies on which the present study is based | 17 |
| 19 | Data and data sharing | Provide the data used to perform all analyses or report where and how the data can be accessed, and reference these sources in the article. Provide the statistical code needed to reproduce the results in the article, or report whether the code is publicly accessible and if so, where | 17 |
| 20 | Conflicts of Interest | All authors should declare all potential conflicts of interest | 17 |

**Table S3. Overview of selected genetic variants and the associations between genetic variant and outcome**

*< attached excel file: ‘Supplementary Table S3.xlsx >*

**Table S4. Selection process and F-statistics of instrumental variables used in Mendelian randomisation analyses**

| Exposure | Sex | Number of SNPs identified by GWAS | Number of SNPs with p-value < 5×10-8 | Number of SNPs after clumping to r^2^ 0.001 | Number of SNPs INFO score > 0.9 | Number of SNPs in HWE | F-statistic |
| --- | --- | --- | --- | --- | --- | --- | --- |
| Diabetes | Females | 14,415,601 | 1,609 | 33 | 32 | 32 | 25.1 |
|  | Males | 14,200,800 | 2,467 | 104 | 101 | 100 | 19.5 |
| HbA1c | Females | 33,811,879 | 419 | 75 | 74 | 73 | 142.2 |
|  | Males | 33,811,879 | 419 | 75 | 74 | 73 | 112.3 |
| Glucose | Females | 8,633,946 | 763 | 24 | 24 | 24 | 88.5 |
|  | Males | 8,637,159 | 579 | 17 | 17 | 17 | 103.9 |
| Insulin | Females | 8,626,268 | 26 | 6 | 6 | 6 | 36.1 |
|  | Males | 8,628,079 | 70 | 4 | 4 | 4 | 38.6 |

Abbreviations: GWAS, genome-wide association study; HWE, Hardy-Weinberg equilibrium; SNP, single nucleotide polymorphism. For diabetes, we report the F-statistic from ordinary least squares regression analysis. For HbA1c, we report the F-statistic from ordinary least squares regression analysis. For glucose and insulin, we report the F-statistic calculated using the formula F = (R^2^ / k) × ((n-k-1) / (1-R^2^)). For more details, see **Supplemental Methods**.

**Table S5. Characteristics of non-diabetic study population in observational analysis**

| **Characteristics** | **No. of non-missing values in females** | **Females (n = 251,941)** | **No. of non-missing values in males** | **Males (n = 193,802)** |
| --- | --- | --- | --- | --- |
| Age, years | 251,941 | 56.1 (8.0) | 193,802 | 56.0 (8.2) |
| Ethnicity | 251,269 |  | 192,997 |  |
| White |  | 238,788 (94.8) |  | 183,449 (94.7) |
| Other* |  | 12,481 (5.0) |  | 9,548 (4.9) |
| Systolic blood pressure, mm Hg | 237,021 | 135.0 (19.3) | 182,509 | 141.0 (17.4) |
| Total cholesterol, mmol/L | 235,035 | 5.9 (1.1) | 182,184 | 5.7 (1.1) |
| HbA1c, mmol/mol | 233,510 | 35.1 (4.2) | 181,007 | 35.1 (4.8) |
| Smoking status | 251,077 |  | 193,137 |  |
| Never smoker |  | 150,786 (59.8) |  | 98,759 (51.0) |
| Former smoker |  | 78,153 (31.0) |  | 70,360 (36.3) |
| Current smoker |  | 22,138 (8.8) |  | 24,018 (12.4) |
| Body mass index, kg/m2 | 250,888 | 26.8 (5.0) | 192,802 | 27.5 (4.0) |
| Socioeconomic status | 251,645 |  | 193,552 |  |
| Townsend deprivation index score |  | -2.21 [-3.7, 0.3] |  | -2.22 [-3.7, 0.4] |
| Townsend deprivation thirds |  |  |  |  |
| Low (≥1.40) |  | 46,083 (18.3) |  | 36,799 (19.0) |
| Middle (≥–2.08 - <1.40) |  | 75,512 (30.0) |  | 56,521 (29.2) |
| High (<–2.08) |  | 130,050 (51.6) |  | 100,232 (51.7) |
| Type 2 diabetes | 251,941 | 0 (0.0) | 193,802 | 0 (0.0) |
| Drug use | 248,894 |  | 190,586 |  |
| Antihypertensive drugs |  | 36,524 (14.5) |  | 33,732 (17.4) |
| Lipid-lowering drugs |  | 21,185 (8.4) |  | 25,790 (13.3) |
| Outcomes |  |  |  |  |
| Cardiovascular disease |  | 17,374 (6.9) |  | 25,462 (13.1) |
| Coronary heart disease |  | 13,053 (5.2) |  | 20,720 (10.7) |
| Myocardial infarction |  | 2,922 (1.2) |  | 6,198 (3.2) |
| Stroke |  | 4,321 (1.7) |  | 4,742 (2.4) |
| Ischaemic stroke |  | 3,131 (1.2) |  | 3,893 (2.0) |
| Intracerebral haemorrhage |  | 678 (0.3) |  | 606 (0.3) |
| Subarachnoid haemorrhage |  | 512 (0.2) |  | 243 (0.1) |

Numbers are presented as mean (standard deviation), median [25^th^, 75^th^ percentile], or number (percentage). *includes Asian or Asian British, Indian, Pakistani, Bangladeshi, any other Asian background, Chinese, black or black British, Caribbean, African, any other black background, other ethnic group, white and black Caribbean, white and black African, white and Asian, and any other mixed background.

**Table S6. Characteristics of study population in Mendelian randomisation analysis**

| **Characteristics** | **No. of non-missing values in females** | **Females (n = 181,183)** | **No. of non-missing values in males** | **Males (n = 156,203)** |
| --- | --- | --- | --- | --- |
| Age, years | 181,183 | 56.7 (7.9) | 156,203 | 57.1 (8.1) |
| Ethnicity | 181,183 |  | 156,203 |  |
| White |  | 181,183 (100.0) |  | 156,203 (100.0) |
| Other* |  | 0 (0.0) |  | 0 (0.0) |
| Systolic blood pressure, mm Hg | 170,529 | 135.6 (19.2) | 147,395 | 141.3 (17.4) |
| Total cholesterol, mmol/L | 172,656 | 5.9 (1.1) | 149,006 | 5.5 (1.1) |
| HbA1c, mmol/mol | 172,652 | 35.7 (5.7) | 148,870 | 36.3 (7.3) |
| Smoking status | 180,564 |  | 155,647 |  |
| Never smoker |  | 107,384 (59.3) |  | 76,390 (48.9) |
| Former smoker |  | 57,607 (31.8) |  | 60,864 (39.0) |
| Current smoker |  | 15,573 (8.6) |  | 18,393 (11.8) |
| Body mass index, kg/m^2^ | 180,634 | 27.0 (5.1) | 155,659 | 27.8 (4.2) |
| Socioeconomic status | 180,971 |  | 156,016 |  |
| Townsend deprivation index score |  | -2.36 [-3.7, 0.0] |  | -2.36 [-3.8, 0.1] |
| Townsend deprivation thirds: |  |  |  |  |
| Low (≥1.40) |  | 29,454 (16.3) |  | 27,015 (17.3) |
| Middle (≥–2.08 - <1.40) |  | 53,559 (29.6) |  | 44,810 (28.7) |
| High (<–2.08) |  | 97,958 (54.1) |  | 84,191 (53.9) |
| Type 2 diabetes | 180,884 | 6,199 (3.4) | 155,809 | 10,051 (6.4) |
| Age at diagnosis of type 2 diabetes, years | 2,876 | 55.1 (8.0) | 5,142 | 55.9 (7.4) |
| Drug use |  |  |  |  |
| Antihypertensive drugs | 180,320 | 31,529 (17.4) | 154,765 | 38,558 (24.7) |
| Lipid-lowering drugs | 180,320 | 22,732 (12.5) | 154,765 | 35,864 (23.0) |
| Outcomes (first occurrence either before or after study entry) |  |  |  |  |
| Cardiovascular disease |  | 19,927 (11.0) |  | 34,610 (22.2) |
| Coronary heart disease |  | 15,445 (8.5) |  | 29,375 (18.8) |
| Myocardial infarction |  | 4,295 (2.4) |  | 12,653 (8.1) |
| Stroke |  | 5,952 (3.3) |  | 8,283 (5.3) |
| Ischaemic stroke |  | 4,808 (2.7) |  | 7,352 (4.7) |
| Intracerebral haemorrhage |  | 802 (0.4) |  | 1,003 (0.6) |
| Subarachnoid haemorrhage |  | 868 (0.5) |  | 551 (0.4) |
| Outcomes (first occurrence before study entry) |  |  |  |  |
| Cardiovascular disease |  | 7,284 (4.0) |  | 15,027 (9.6) |
| Coronary heart disease |  | 5,707 (3.1) |  | 13,114 (8.4) |
| Myocardial infarction |  | 1,746 (1.0) |  | 7,192 (4.6) |
| Stroke |  | 2,496 (1.4) |  | 3,995 (2.6) |
| Ischaemic stroke |  | 2,184 (1.2) |  | 3,704 (2.4) |
| Intracerebral haemorrhage |  | 165 (0.1) |  | 350 (0.2) |
| Subarachnoid haemorrhage |  | 384 (0.2) |  | 308 (0.2) |

Numbers are presented as mean (standard deviation), median [25^th^, 75^th^ percentile], or number (percentage). *includes Asian or Asian British, Indian, Pakistani, Bangladeshi, any other Asian background, Chinese, black or black British, Caribbean, African, any other black background, other ethnic group, white and black Caribbean, white and black African, white and Asian, and any other mixed background.

**Table S7. Characteristics of non-diabetic study population in Mendelian randomisation analysis**

| **Characteristics** | **No. of non-missing values in females** | **Females (n = 174,685)** | **No. of non-missing values in males** | **Males (n = 145,758)** |
| --- | --- | --- | --- | --- |
| Age, years | 174,685 | 56.6 (7.9) | 145,758 | 56.9 (8.1) |
| Ethnicity | 174,685 |  | 145,758 |  |
| White |  | 174,685 (100.0) |  | 145,758 (100.0) |
| Other* |  | 0 (0.0) |  | 0 (0.0) |
| Systolic blood pressure, mm Hg | 164,453 | 135.5 (19.2) | 137,512 | 141.2 (17.5) |
| Total cholesterol, mmol/L | 166,490 | 5.9 (1.1) | 139,045 | 5.6 (1.1) |
| HbA1c, mmol/mol | 166,443 | 35.1 (4.1) | 138,867 | 35.1 (4.9) |
| Smoking status | 174,102 |  | 145,282 |  |
| Never smoker |  | 103,874 (59.5) |  | 72,607 (49.8) |
| Former smoker |  | 55,266 (31.6) |  | 55,526 (38.1) |
| Current smoker |  | 14,962 (8.6) |  | 17,149 (11.8) |
| Body mass index, kg/m^2^ | 174,185 | 26.8 (5.0) | 145,302 | 27.6 (4.0) |
| Socioeconomic status | 174,482 |  | 145,582 |  |
| Townsend deprivation index score |  | -2.38 [-3.8, -0.1] |  | -2.39 [-3.8, 0.0] |
| Townsend deprivation thirds: |  |  |  |  |
| Low (≥1.40) |  | 27,785 (15.9) |  | 24,369 (16.7) |
| Middle (≥–2.08 - <1.40) |  | 51,529 (29.5) |  | 41,759 (28.6) |
| High (<–2.08) |  | 95,168 (54.5) |  | 79,454 (54.5) |
| Drug use |  |  |  |  |
| Antihypertensive drugs | 173,867 | 27,792 (15.9) | 144,450 | 31,718 (21.8) |
| Lipid-lowering drugs | 173,867 | 18,161 (10.4) | 144,450 | 27,926 (19.2) |
| Outcomes (first occurrence either before or after study entry) |  |  |  |  |
| Cardiovascular disease |  | 18,025 (10.3) |  | 29,954 (20.6) |
| Coronary heart disease |  | 13,809 (7.9) |  | 25,234 (17.3) |
| Myocardial infarction |  | 3,791 (2.2) |  | 10,720 (7.4) |
| Stroke |  | 5,430 (3.1) |  | 7,099 (4.9) |
| Ischaemic stroke |  | 4,328 (2.5) |  | 6,251 (4.3) |
| Intracerebral haemorrhage |  | 756 (0.4) |  | 876 (0.6) |
| Subarachnoid haemorrhage |  | 834 (0.5) |  | 509 (0.3) |
| Outcomes (first occurrence before study entry) |  |  |  |  |
| Cardiovascular disease |  | 6,347 (3.6) |  | 12,360 (8.5) |
| Coronary heart disease |  | 4,881 (2.8) |  | 10,663 (7.3) |
| Myocardial infarction |  | 1,437 (0.8) |  | 5,764 (4.0) |
| Stroke |  | 2,207 (1.3) |  | 3,266 (2.2) |
| Ischaemic stroke |  | 1,910 (1.1) |  | 3,004 (2.1) |
| Intracerebral haemorrhage |  | 146 (0.1) |  | 288 (0.2) |
| Subarachnoid haemorrhage |  | 367 (0.2) |  | 283 (0.2) |

Numbers are presented as mean (standard deviation), median [25^th^, 75^th^ percentile], or number (percentage). *includes Asian or Asian British, Indian, Pakistani, Bangladeshi, any other Asian background, Chinese, black or black British, Caribbean, African, any other black background, other ethnic group, white and black Caribbean, white and black African, white and Asian, and any other mixed background.

**Table S8. Q test results**

|  |  | **Q-test IVW** | |
| --- | --- | --- | --- |
|  |  |  |  |
| **Outcome / Exposure** | **Sex** | **Q** | **P-value** |
| **Diabetes** | | | |
| Cardiovascular disease | Females | 38.0 | 0.180 |
|  | Males | 385.5 | <0.001 |
| Coronary heart disease | Females | 36.5 | 0.230 |
|  | Males | 403.5 | <0.001 |
| Myocardial infarction | Females | 27.5 | 0.646 |
|  | Males | 298.9 | <0.001 |
| Stroke | Females | 27.1 | 0.665 |
|  | Males | 125.2 | 0.038 |
| Ischaemic stroke | Females | 30.3 | 0.504 |
|  | Males | 119.0 | 0.083 |
| Intracerebral haemorrhage | Females | 30.1 | 0.513 |
|  | Males | 105.0 | 0.321 |
| Subarachnoid haemorrhage | Females | 33.0 | 0.369 |
|  | Males | 107.3 | 0.266 |
| **HbA1c** |  |  |  |
| Cardiovascular disease | Females | 130.9 | <0.001 |
|  | Males | 212.6 | <0.001 |
| Coronary heart disease | Females | 126.9 | <0.001 |
|  | Males | 204.6 | <0.001 |
| Myocardial infarction | Females | 124.5 | <0.001 |
|  | Males | 192.3 | <0.001 |
| Stroke | Females | 81.1 | 0.217 |
|  | Males | 120.3 | <0.001 |
| Ischaemic stroke | Females | 101.5 | 0.013 |
|  | Males | 113.3 | 0.001 |
| Intracerebral haemorrhage | Females | 81.8 | 0.201 |
|  | Males | 59.4 | 0.857 |
| Subarachnoid haemorrhage | Females | 87.7 | 0.101 |
|  | Males | 70.5 | 0.528 |

**Table S9. MR-PRESSO**

|  |  | **Primary analysis**  **(IVW)** |  | | **MR-PRESSO** | | | |
| --- | --- | --- | --- | --- | --- | --- | --- | --- |
|  |  |  |  |  | | | | |
| **Outcome / Exposure** | **Sex** | **OR (95% CI)** |  | **Global p-value** | | **Outliers detected** | **OR (95% CI)** | **p-value distortion test *** |
| **Diabetes** | | |  |  | |  |  |  |
| Cardiovascular disease | Females | 1.07 (1.03, 1.12) |  | 0.214 | | – | – | – |
|  | Males | 1.10 (1.05, 1.15) |  | <0.001 | | ✓ | 1.10 (1.07, 1.14) | 0.902 |
| Coronary heart disease | Females | 1.08 (1.03, 1.13) |  | 0.253 | | – | – | – |
|  | Males | 1.11 (1.05, 1.16) |  | <0.001 | | ✓ | 1.10 (1.07, 1.14) | 0.908 |
| Myocardial infarction | Females | 1.12 (1.03, 1.21) |  | 0.648 | | – | – | – |
|  | Males | 1.12 (1.05, 1.19) |  | <0.001 | | ✓ | 1.12 (1.07, 1.18) | 0.894 |
| Stroke | Females | 1.04 (0.97, 1.12) |  | 0.688 | | – | – | – |
|  | Males | 1.05 (1.00, 1.10) |  | 0.035 | | – | – | – |
| Ischaemic stroke | Females | 1.06 (0.98, 1.14) |  | 0.541 | | – | – | – |
|  | Males | 1.06 (1.01, 1.12) |  | 0.086 | | – | – | – |
| Intracerebral haemorrhage | Females | 1.10 (0.91, 1.32) |  | 0.516 | | – | – | – |
|  | Males | 1.01 (0.90, 1.14) |  | 0.316 | | – | – | – |
| Subarachnoid haemorrhage | Females | 0.89 (0.74, 1.06) |  | 0.393 | | – | – | – |
|  | Males | 1.04 (0.88, 1.22) |  | 0.271 | | – | – | – |
| **HbA1c** |  |  |  |  | |  |  |  |
| Cardiovascular disease | Females | 1.01 (0.79, 1.29) |  | <0.001 | | ✓ | 1.02 (0.83, 1.25) | 0.937 |
|  | Males | 1.37 (1.06, 1.77) |  | <0.001 | | ✓ | 1.47 (1.21, 1.79) | 0.657 |
| Coronary heart disease | Females | 1.09 (0.83, 1.42) |  | <0.001 | | ✓ | 1.11 (0.89, 1.38) | 0.873 |
|  | Males | 1.48 (1.13, 1.93) |  | <0.001 | | ✓ | 1.59 (1.29, 1.96) | 0.596 |
| Myocardial infarction | Females | 0.90 (0.55, 1.45) |  | 0.000 | | ✓ | 0.96 (0.62, 1.49) | 0.205 |
|  | Males | 1.28 (0.89, 1.84) |  | <0.001 | | ✓ | 1.73 (1.26, 2.37) | 0.274 |
| Stroke | Females | 0.84 (0.60, 1.17) |  | 0.214 | | – | – | – |
|  | Males | 1.12 (0.79, 1.58) |  | <0.001 | | ✓ | 1.17 (0.84, 1.62) | 0.859 |
| Ischaemic stroke | Females | 0.93 (0.61, 1.40) |  | 0.012 | | ✓ | 0.97 (0.66, 1.44) | 0.251 |
|  | Males | 1.14 (0.79, 1.62) |  | 0.001 | | ✓ | 1.20 (0.86, 1.66) | 0.829 |
| Intracerebral haemorrhage | Females | 0.57 (0.23, 1.40) |  | 0.212 | | – | – | – |
|  | Males | 1.69 (0.80, 3.58) |  | 0.865 | | – | – | – |
| Subarachnoid haemorrhage | Females | 0.44 (0.18, 1.08) |  | 0.112 | | – | – | – |
|  | Males | 0.84 (0.31, 2.33) |  | 0.549 | | – | – | – |
| **Glucose** | | |  |  | |  |  |  |
| Cardiovascular disease | Females | 1.09 (0.94, 1.26) |  | 0.335 | | – | – | – |
|  | Males | 1.13 (0.99, 1.29) |  | 0.016 | | ✓ | 1.16 (1.03, 1.30) | 0.638 |
| Coronary heart disease | Females | 1.13 (0.96, 1.33) |  | 0.384 | | – | – | – |
|  | Males | 1.16 (1.01, 1.32) |  | 0.044 | | ✓ | 1.19 (1.06, 1.32) | 0.613 |
| Myocardial infarction | Females | 1.25 (0.93, 1.66) |  | 0.395 | | – | – | – |
|  | Males | 1.13 (0.97, 1.32) |  | 0.204 | | – | – | – |
| Stroke | Females | 1.03 (0.79, 1.35) |  | 0.135 | | – | – | – |
|  | Males | 1.06 (0.88, 1.27) |  | 0.229 | | – | – | – |
| Ischaemic stroke | Females | 1.08 (0.78, 1.51) |  | 0.024 | | ✓ | 0.90 (0.65, 1.24) | 0.245 |
|  | Males | 1.04 (0.86, 1.25) |  | 0.234 | | – | – | – |
| Intracerebral haemorrhage | Females | 0.83 (0.43, 1.62) |  | 0.338 | | – | – | – |
|  | Males | 1.25 (0.82, 1.91) |  | 0.559 | | – | – | – |
| Subarachnoid haemorrhage | Females | 0.75 (0.36, 1.54) |  | 0.117 | | – | – | – |
|  | Males | 0.91 (0.47, 1.76) |  | 0.282 | | – | – | – |
| **Insulin** | | |  |  | |  |  |  |
| Cardiovascular disease | Females | 1.07 (0.92, 1.26) |  | 0.752 | | – | – | – |
|  | Males | 1.60 (0.70, 3.67) |  | 0.035 | | ✓ | 1.65 (1.37, 1.99) | NA |
| Coronary heart disease | Females | 1.03 (0.82, 1.30) |  | 0.328 | | – | – | – |
|  | Males | 1.58 (0.63, 3.96) |  | 0.037 | | ✓ | 1.90 (1.70, 2.12) | NA |
| Myocardial infarction | Females | 1.18 (0.79, 1.78) |  | 0.317 | | – | – | – |
|  | Males | 2.20 (0.82, 5.94) |  | 0.088 | | – | – | – |
| Stroke | Females | 1.18 (0.90, 1.55) |  | 0.525 | | – | – | – |
|  | Males | 1.83 (1.05, 3.19) |  | 0.985 | | – | – | – |
| Ischaemic stroke | Females | 1.21 (0.90, 1.64) |  | 0.770 | | – | – | – |
|  | Males | 1.85 (1.03, 3.32) |  | 0.958 | | – | – | – |
| Intracerebral haemorrhage | Females | 0.88 (0.29, 2.68) |  | 0.209 | | – | – | – |
|  | Males | 4.57 (0.51, 40.88) |  | 0.239 | | – | – | – |
| Subarachnoid haemorrhage | Females | 1.74 (0.80, 3.80) |  | 0.379 | | – | – | – |
|  | Males | 0.68 (0.08, 5.62) |  | 0.482 | | – | – | – |

*The distortion test tests the difference between the estimate before and after removal of detected outlier SNPs. Abbreviations: CI, confidence interval; IVW, inverse-variance weighting; MR-PRESSO, MR Pleiotropy Residual Sum and Outlier; OR, odds ratio; SNP, single nucleotide polymorphism.

**Supplementary Figures**

**Figure S1. Cox regression of the association between diabetes and cardiovascular disease outcomes in females and males, using the same UK Biobank participants as included in Mendelian randomisation analyses**


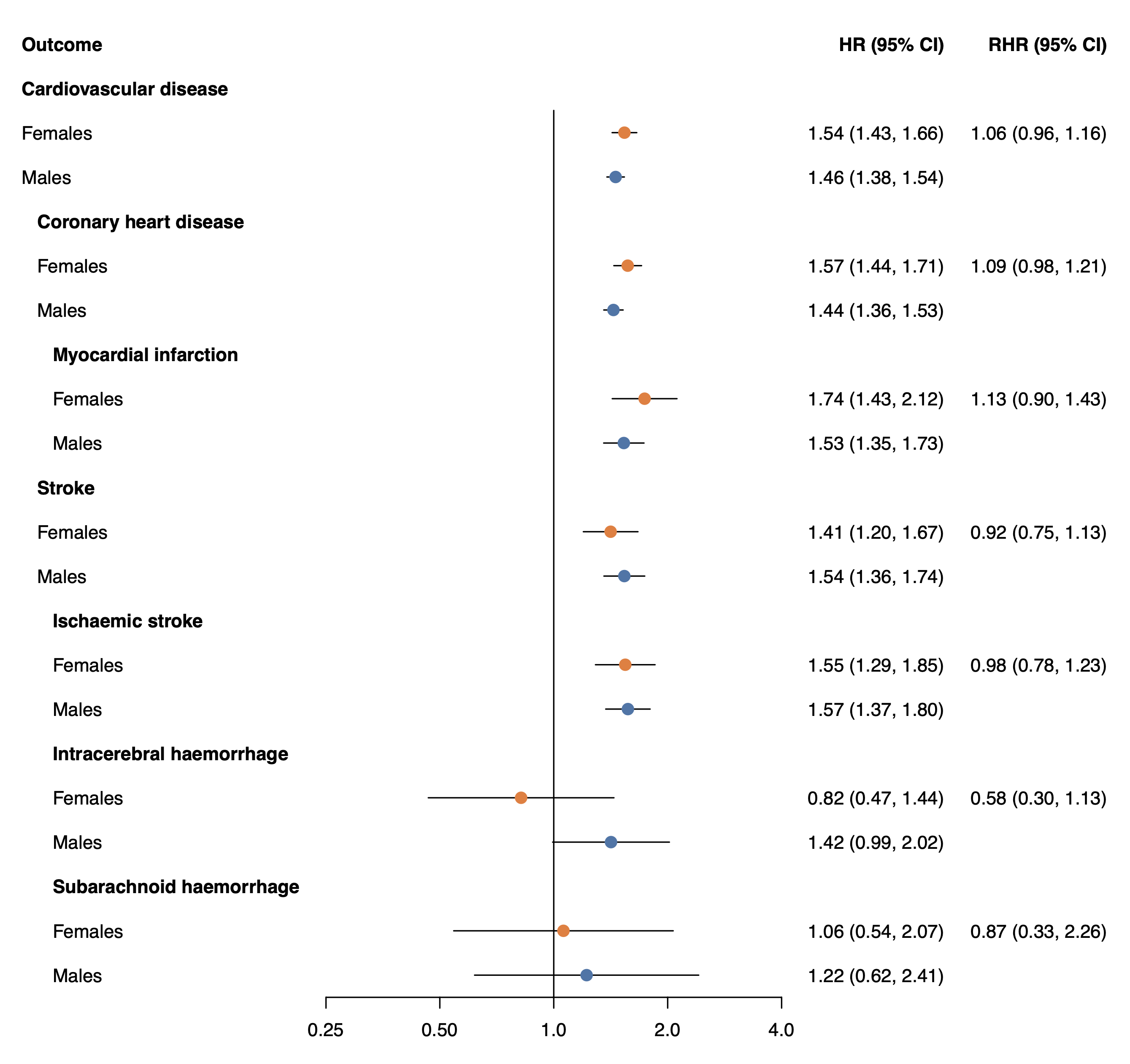
Cox regressions were performed in 315,073 UK Biobank participants and adjusted for sex, Townsend deprivation index (an area-based measure of socioeconomic status), systolic blood pressure, total cholesterol levels, smoking status, body mass index, use of lipid lowering medication, and use of antihypertensives, including an interaction term between each of these adjustment variables and sex. RHRs present the female-to-male ratios of HRs as obtained from an interaction term of diabetes and sex.
Abbreviations: CI, confidence interval; HR, hazard ratio; RHR, ratio of hazard ratios.

**Figure S2. Cox regression of the association between HbA1c and cardiovascular disease outcomes in females and males, using the same UK Biobank participants as included in Mendelian randomisation analyses**


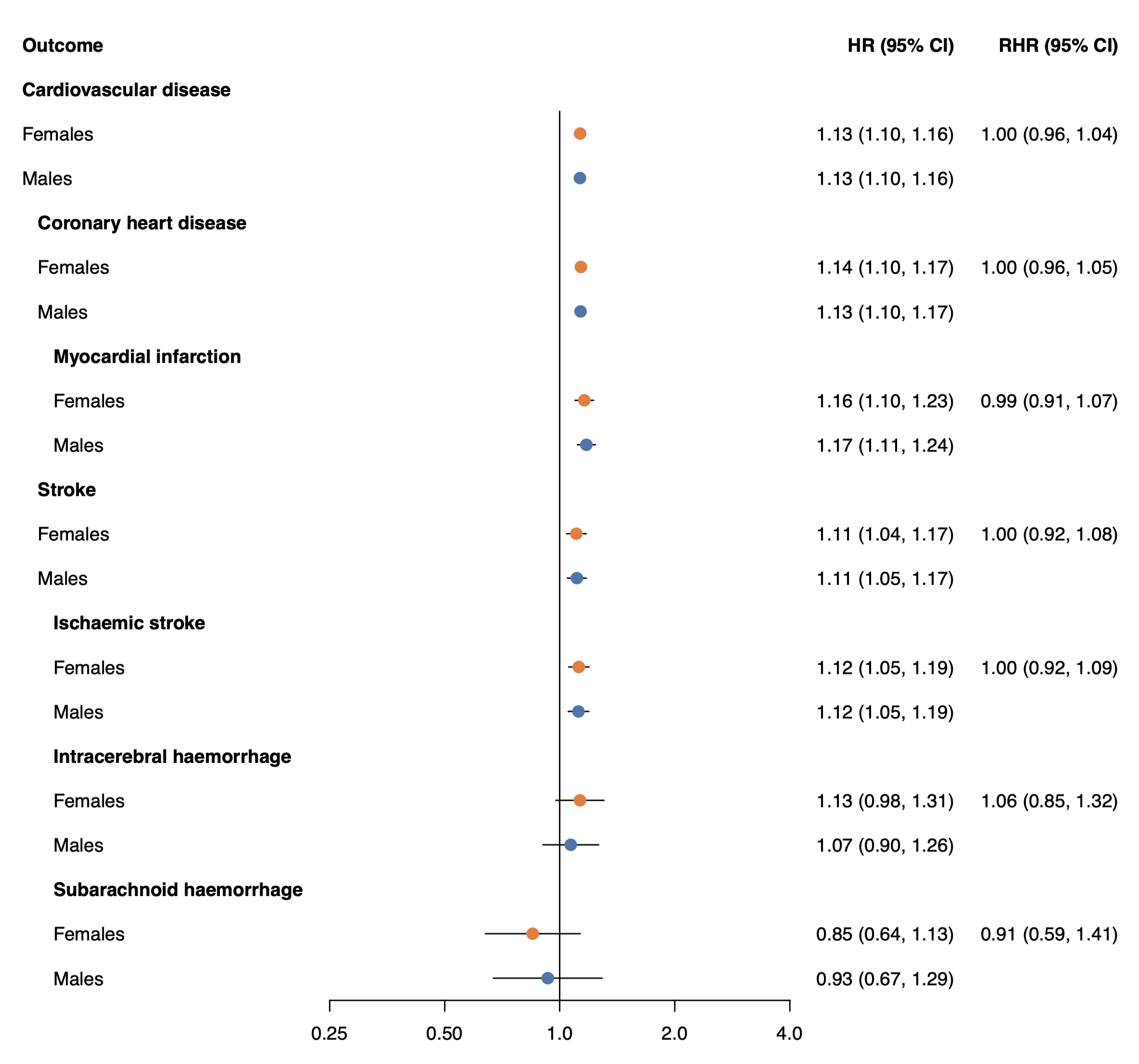
Cox regressions were performed in 315,073 UK Biobank participants and adjusted for sex, type 2 diabetes status, the Townsend deprivation index (an area-based measure of socioeconomic status), systolic blood pressure, total cholesterol levels, smoking status, body mass index, use of lipid lowering medication, and use of antihypertensives, including an interaction term between each of these adjustment variables and sex. RHRs present the female-to-male ratios of HRs as obtained from an interaction term of HbA1c and sex.
Abbreviations: CI, confidence interval; HR, hazard ratio; RHR, ratio of hazard ratios.

**Figure S3. Mendelian randomisation estimates of the association between diabetes and cardiovascular disease outcomes in females based on different methods**

**
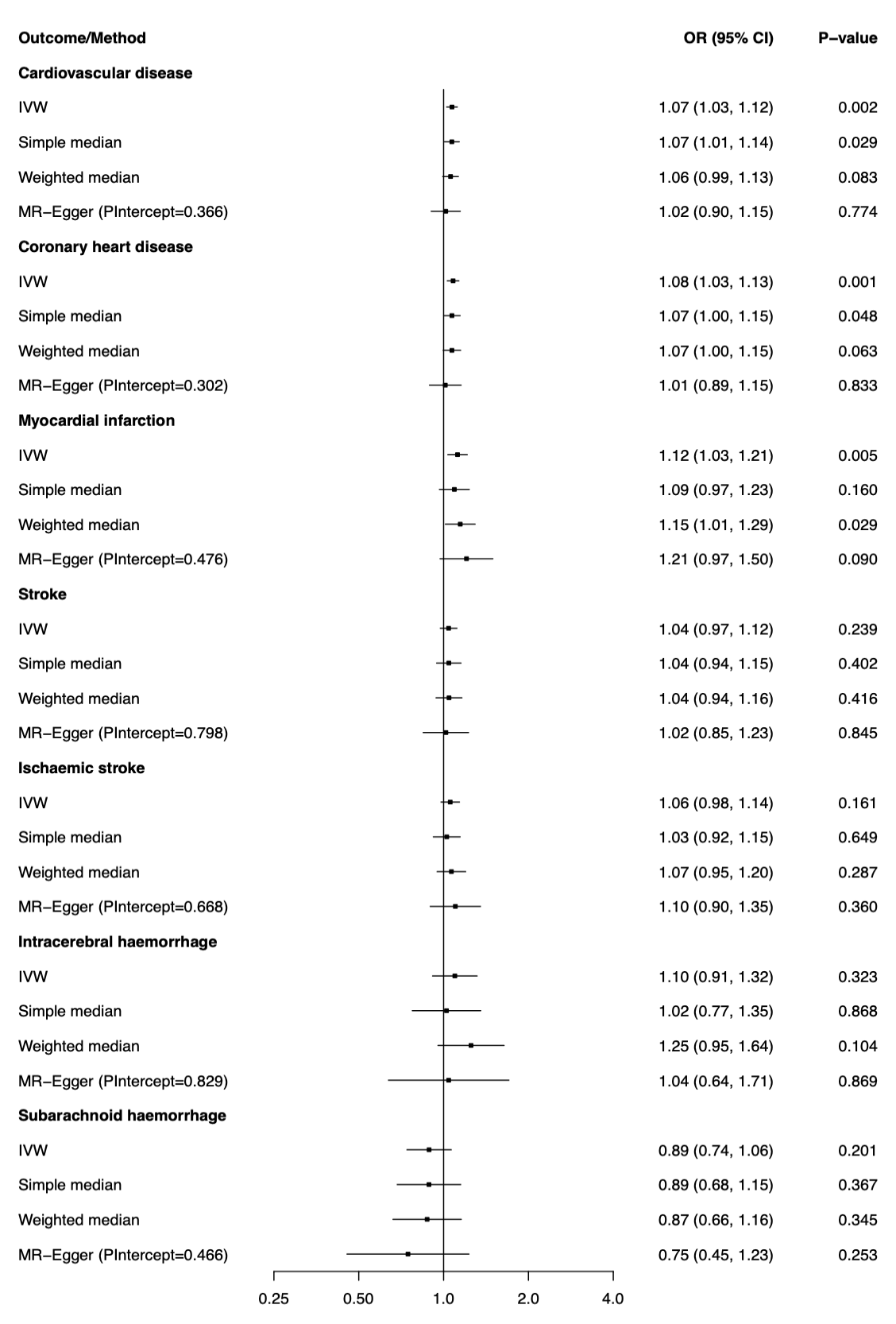
**

Abbreviations: IVW, inverse-variance weighting; MR, Mendelian randomisation.

**Figure S4. Mendelian randomisation estimates of the association between diabetes and cardiovascular disease outcomes in males based on different methods**

**
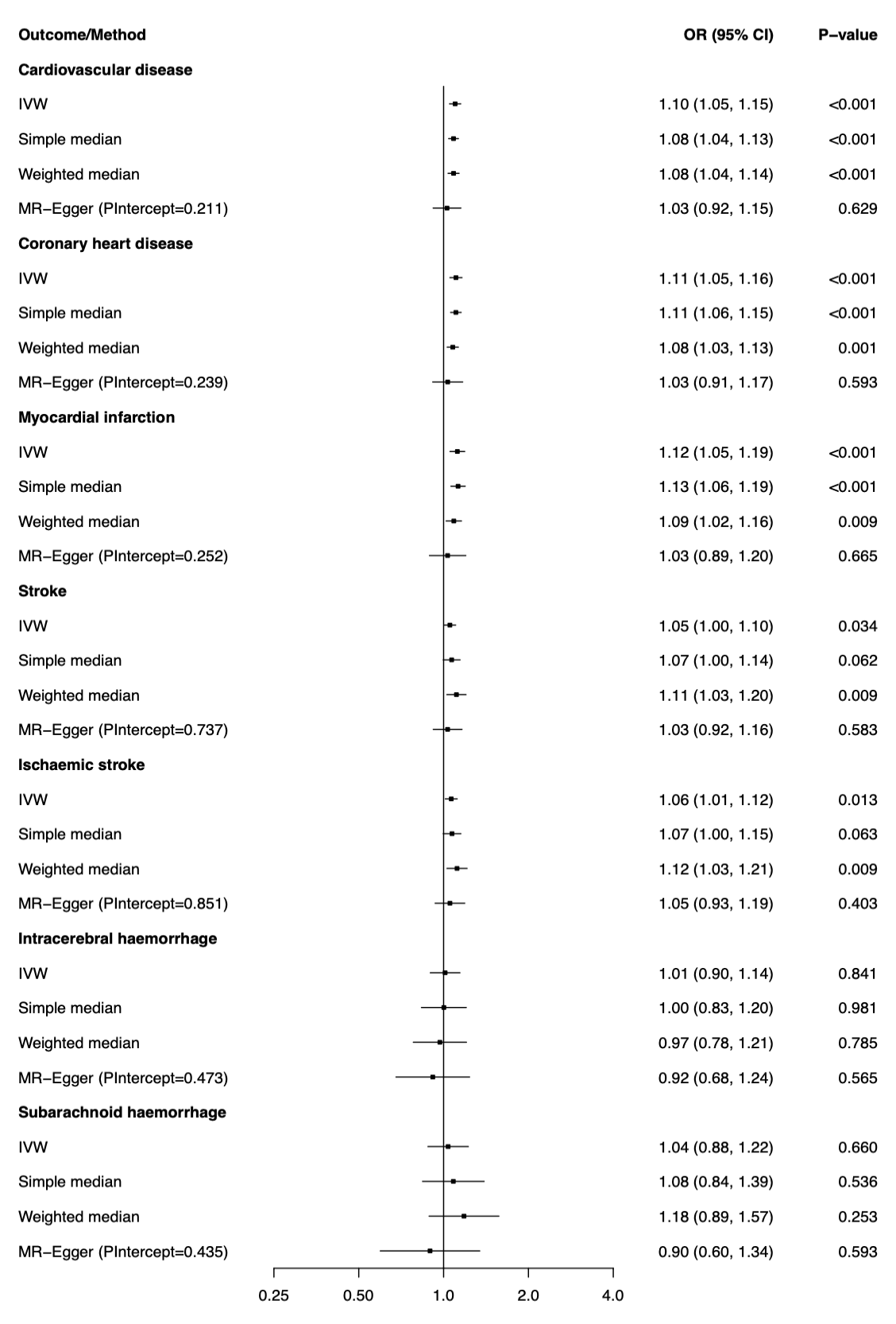
**

Abbreviations: IVW, inverse-variance weighting; MR, Mendelian randomisation.

**Figure S5. Mendelian randomisation estimates of the association between HbA1c and cardiovascular disease outcomes in females based on different methods**

**
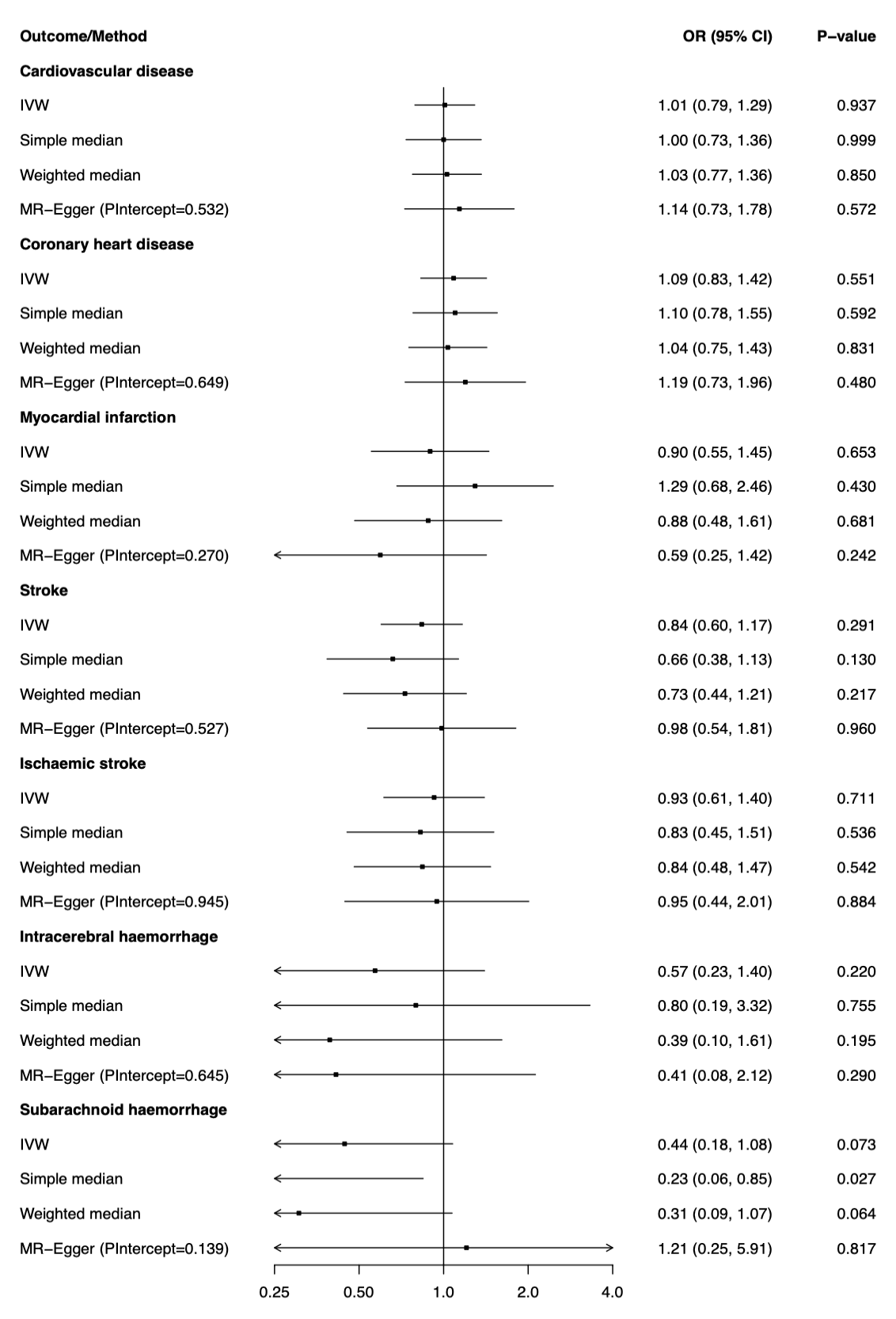
**

Abbreviations: IVW, inverse-variance weighting; MR, Mendelian randomisation.

**Figure S6. Mendelian randomisation estimates of the association between HbA1c and cardiovascular disease outcomes in males based on different methods**

**
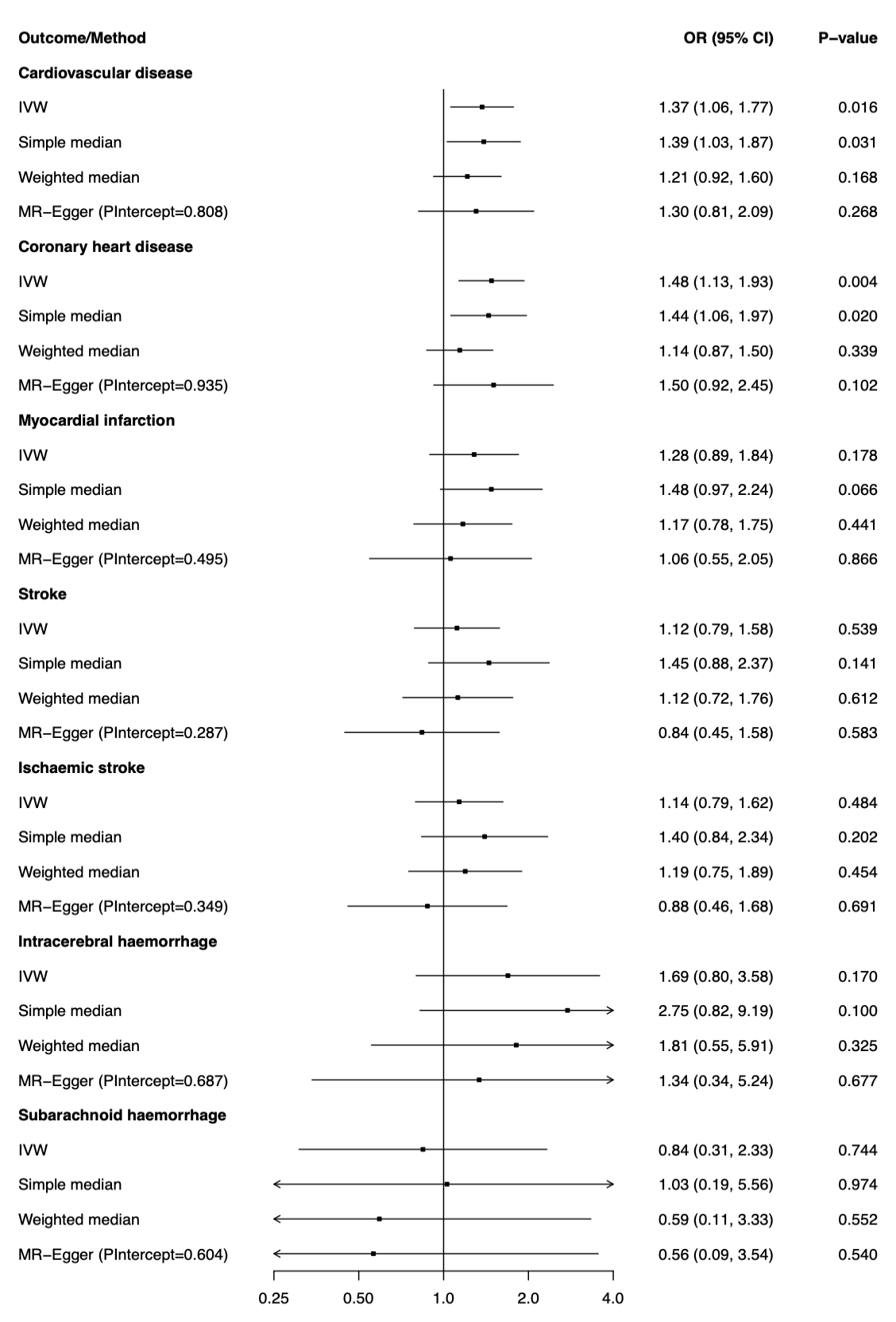
**

Abbreviations: IVW, inverse-variance weighting; MR, Mendelian randomisation.

**Figure S7. Mendelian randomisation estimates of the association between fasting glucose and cardiovascular disease outcomes in females based on different methods**

**
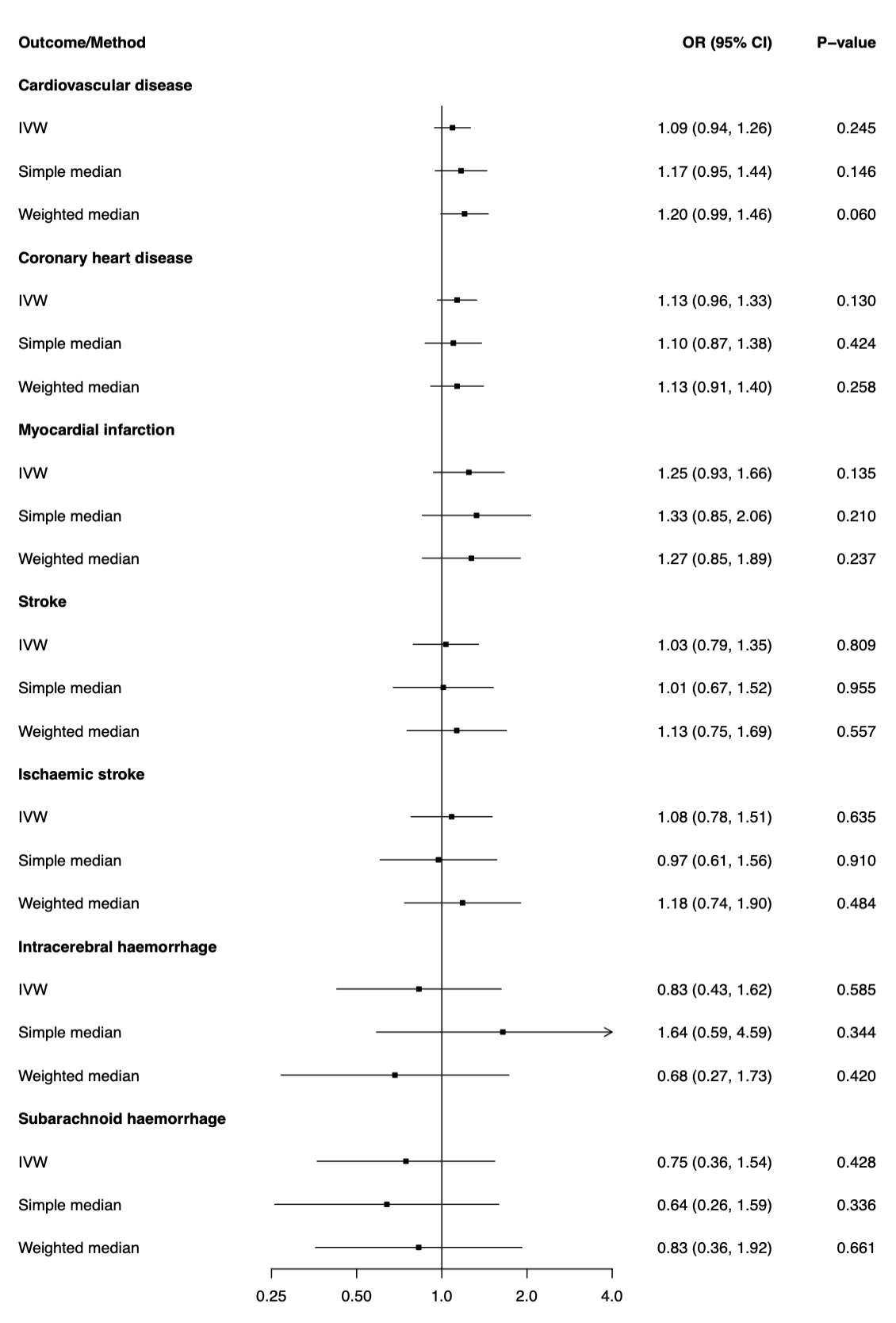
**

Abbreviation: IVW, inverse-variance weighting.

**Figure S8. Mendelian randomisation estimates of the association between fasting glucose and cardiovascular disease outcomes in males based on different methods**

**
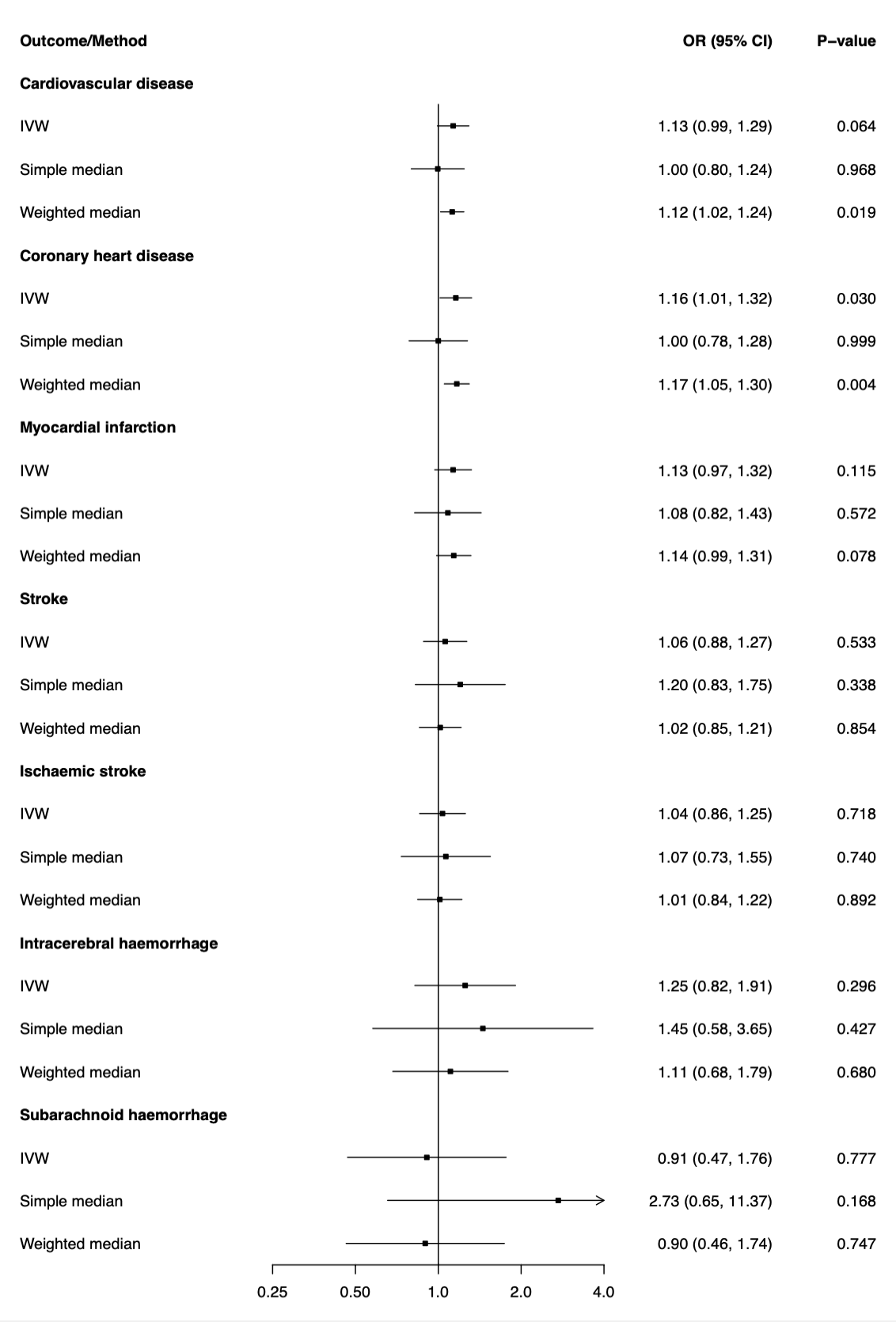
**

Abbreviation: IVW, inverse-variance weighting.

**Figure S9. Mendelian randomisation estimates of the association between fasting insulin and cardiovascular disease outcomes in females based on different methods**

**
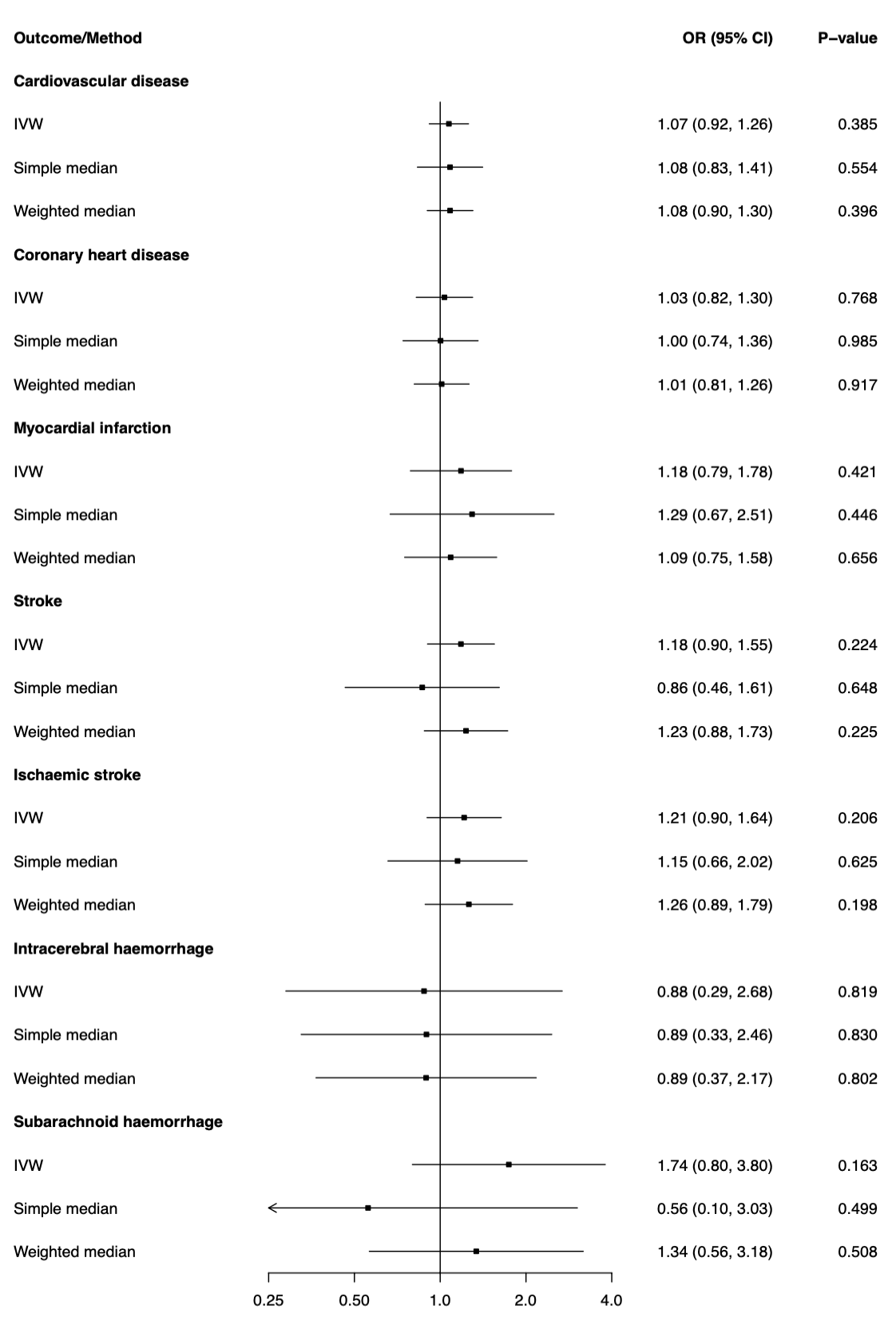
**

Abbreviation: IVW, inverse-variance weighting.

**Figure S10. Mendelian randomisation estimates of the association between fasting insulin and cardiovascular disease outcomes in males based on different methods
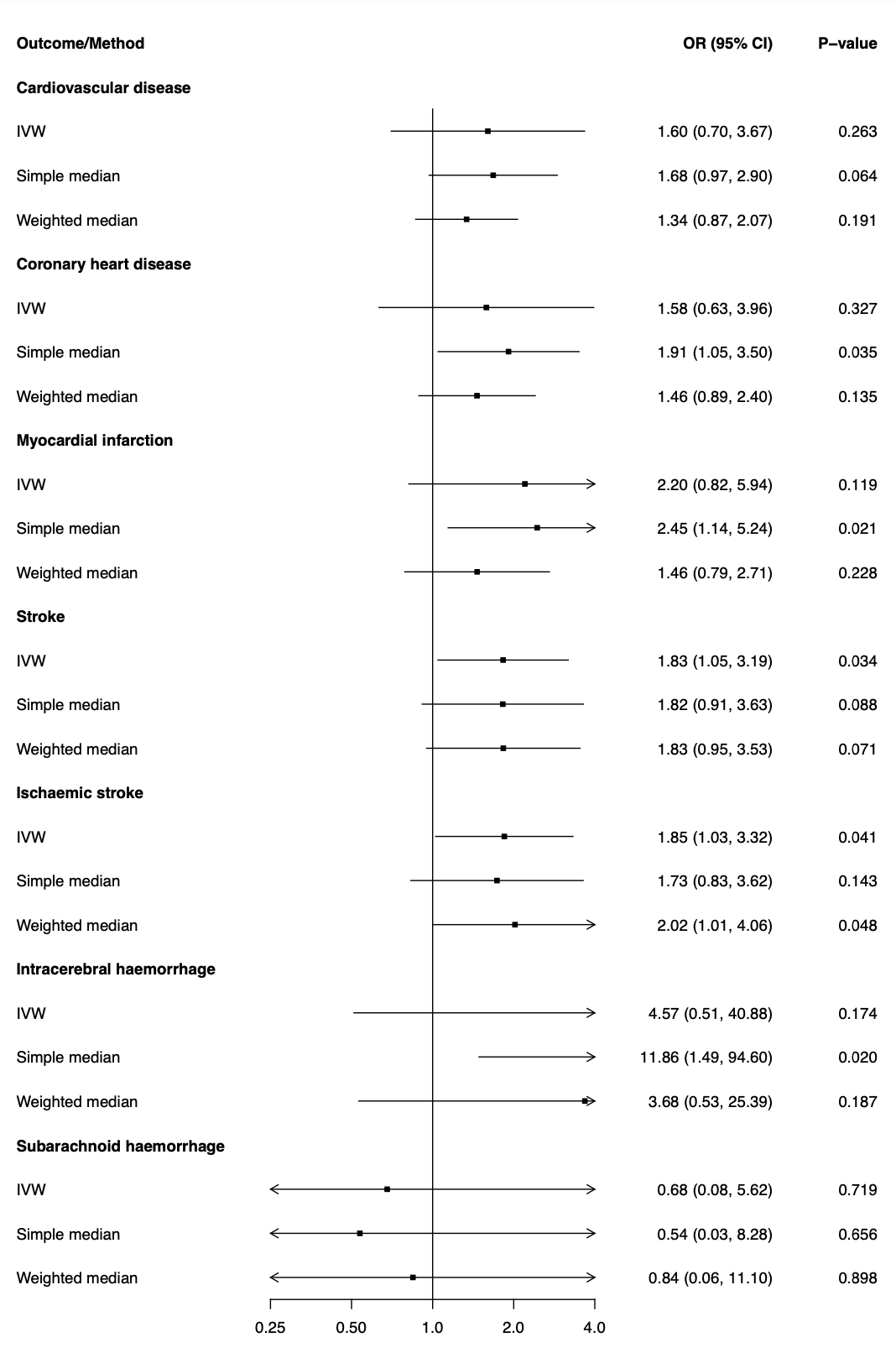
**

Abbreviation: IVW, inverse-variance weighting.

**Figure S11. Mendelian randomisation estimates of the association between HbA1c and cardiovascular disease outcomes in females and males, with adjustment for diabetes status**


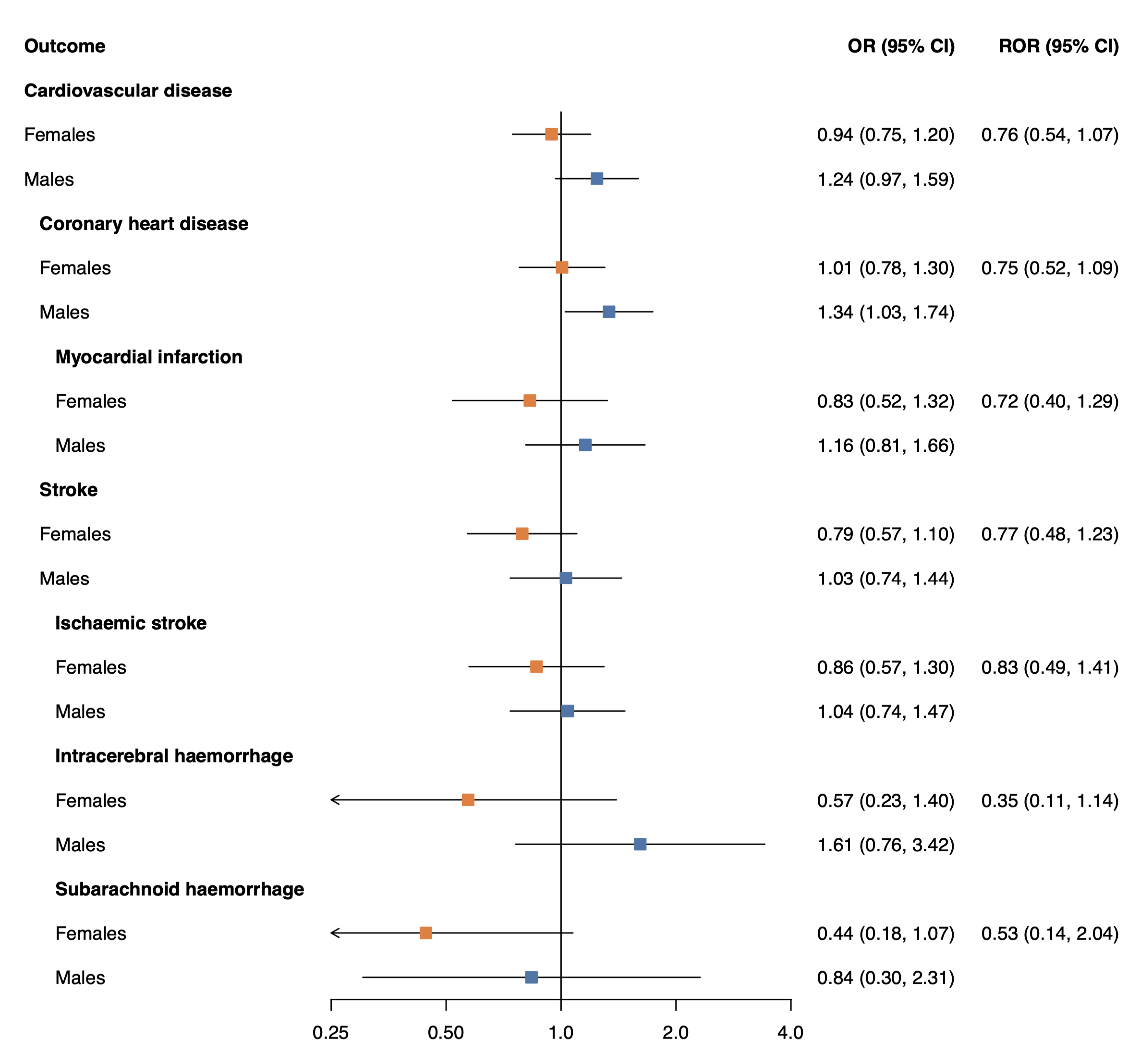
Estimates are from inverse-variance weighted MR. The odds ratios (ORs) can be interpreted as the effect per one percent increase in genetically predicted HbA1c level. MR analyses were performed in 337,386 UK Biobank participants. RORs present the female-to-male ratios of ORs as obtained from two separate MR analyses.
Abbreviations: CI, confidence interval; OR, odds ratio; ROR, ratio of odds ratios.

**Figure S12. Mendelian randomisation estimates of the association between fasting glucose and cardiovascular disease outcomes in females and males, with adjustment for diabetes status**


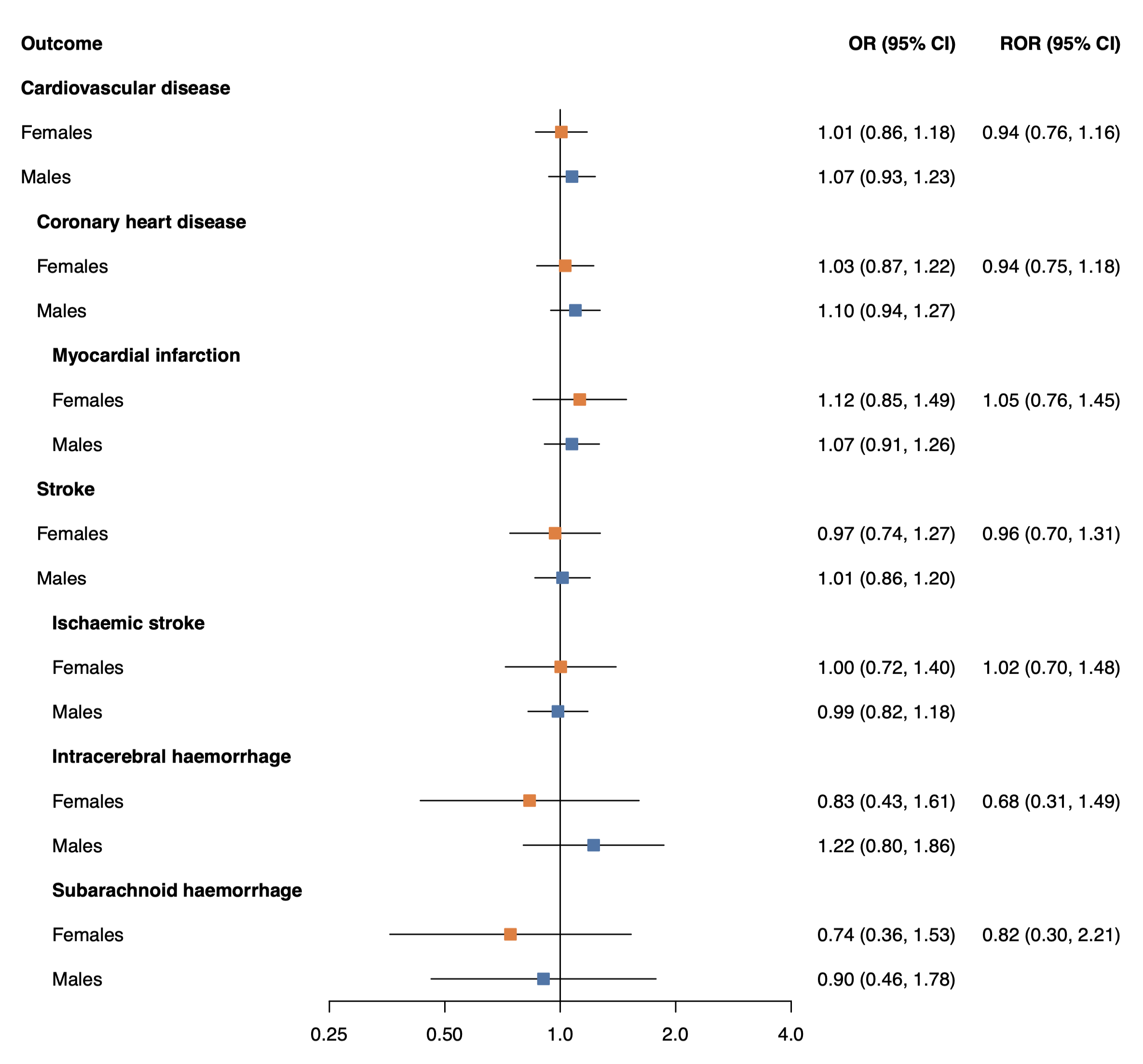
Estimates are from inverse-variance weighted MR. The odds ratios (ORs) can be interpreted as the effect per one mmol/L increase in genetically predicted fasting glucose level. MR analyses were performed in 337,386 UK Biobank participants. RORs present the female-to-male ratios of ORs as obtained from two separate MR analyses. RORs present the female-to-male ratios of ORs as obtained from two separate MR analyses.
Abbreviations: CI, confidence interval; OR, odds ratio; ROR, ratio of odds ratios.

**Figure S13. Mendelian randomisation estimates of the association between fasting insulin and cardiovascular disease outcomes in females and males, with adjustment for diabetes status**

**
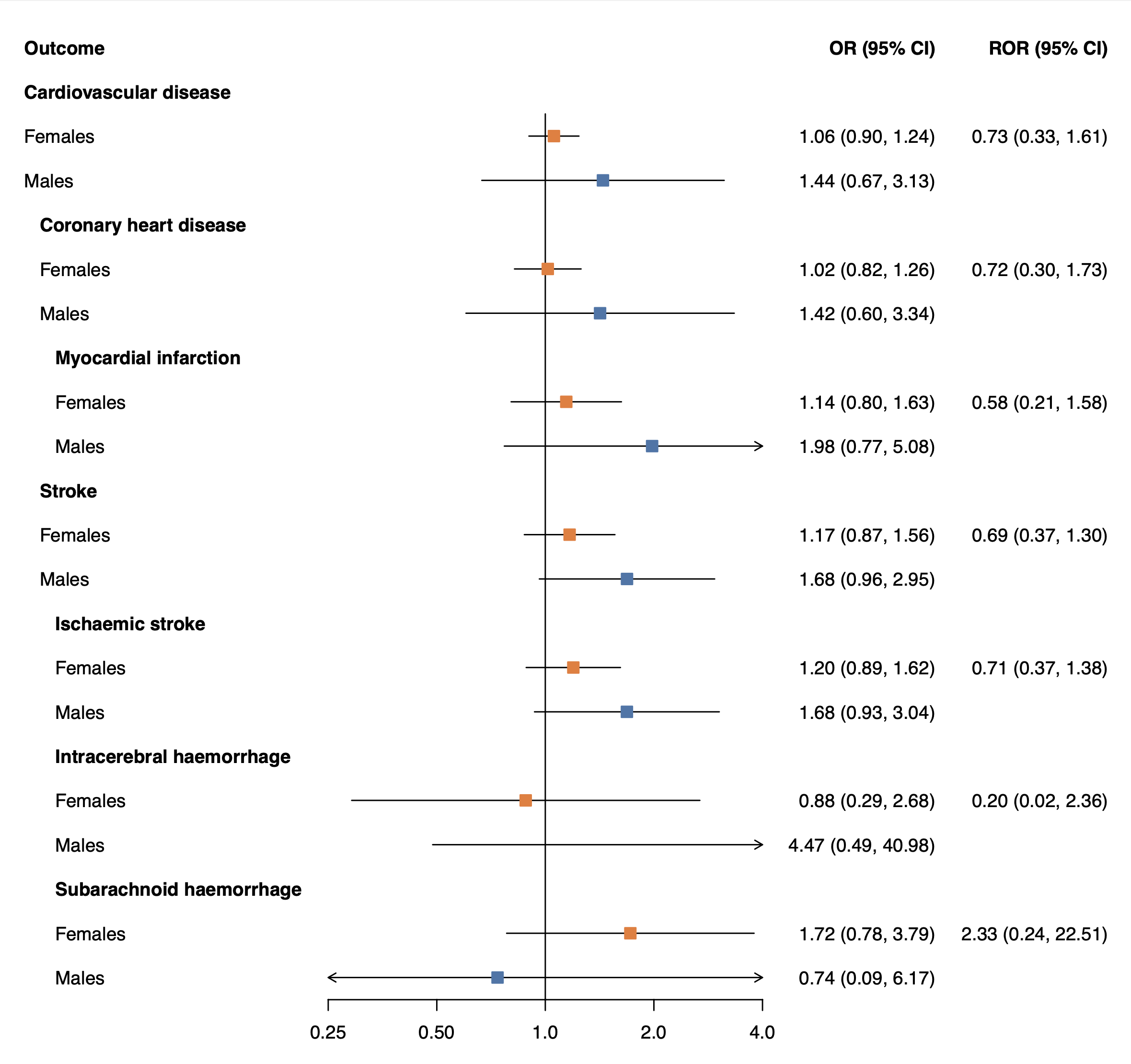
**Estimates are from inverse-variance weighted MR. The odds ratios (ORs) can be interpreted as the effect per one log unit increase in genetically predicted fasting insulin level. MR analyses were performed in 337,386 UK Biobank participants. RORs present the female-to-male ratios of ORs as obtained from two separate MR analyses.
Abbreviations: CI, confidence interval; OR, odds ratio; ROR, ratio of odds ratios.

**Figure S14. Cox regression estimates and Mendelian randomisation estimates of the association between HbA1c and cardiovascular disease outcomes in non-diabetic females and males**

**
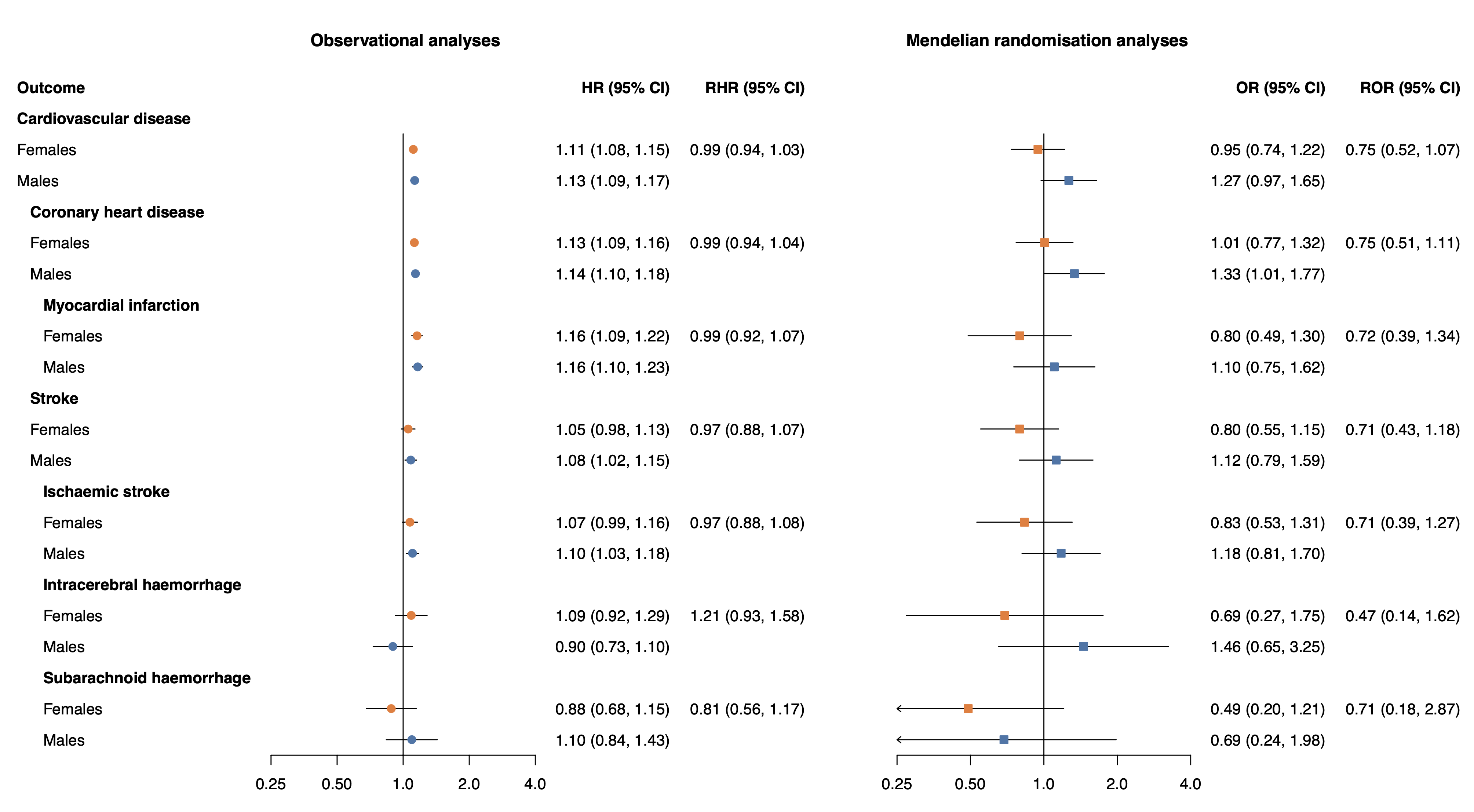
**Mendelian randomisation estimates are from inverse-variance weighted MR and odds ratios (ORs) can be interpreted as the effect per one percent increase in genetically predicted HbA1c level. MR analyses were performed in 320,443 UK Biobank participants. Cox regressions were performed in 445,743 UK Biobank participants and adjusted for sex, Townsend deprivation index (an area-based measure of socioeconomic status), systolic blood pressure, total cholesterol levels, smoking status, body mass index, use of lipid lowering medication, and use of antihypertensives, including an interaction term between each of these adjustment variables and sex. RHRs present the female-to-male ratios of HRs as obtained from an interaction term of HbA1c with sex, and RORs present the female-to-male ratios of ORs as obtained from two separate MR analyses.
Abbreviations: CI, confidence interval; HR, hazard ratio; OR, odds ratio; RHR, ratio of hazard ratios; ROR, ratio of odds ratios.

**Figure S15. Mendelian randomisation estimates of the association between fasting glucose and cardiovascular disease outcomes in non-diabetic females and males**


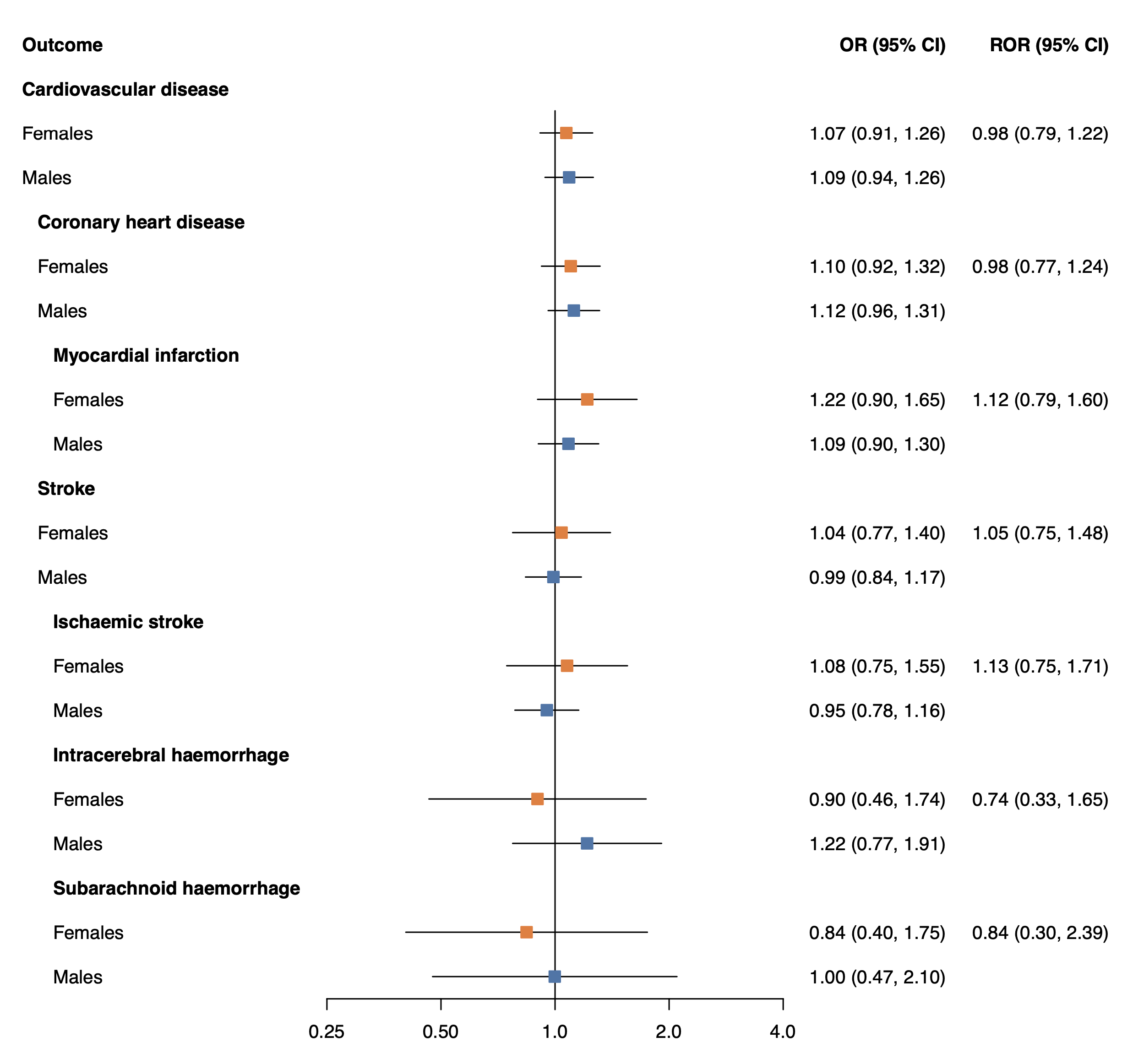
Estimates are from inverse-variance weighted MR. The odds ratios (ORs) can be interpreted as the effect per one mmol/L increase in genetically predicted fasting glucose level. MR analyses were performed in 320,443 UK Biobank participants. RORs present the female-to-male ratios of ORs as obtained from two separate MR analyses.
Abbreviations: CI, confidence interval; OR, odds ratio; ROR, ratio of odds ratios.

**Figure S16. Mendelian randomisation estimates of the association between fasting insulin and cardiovascular disease outcomes in non-diabetic females and males**


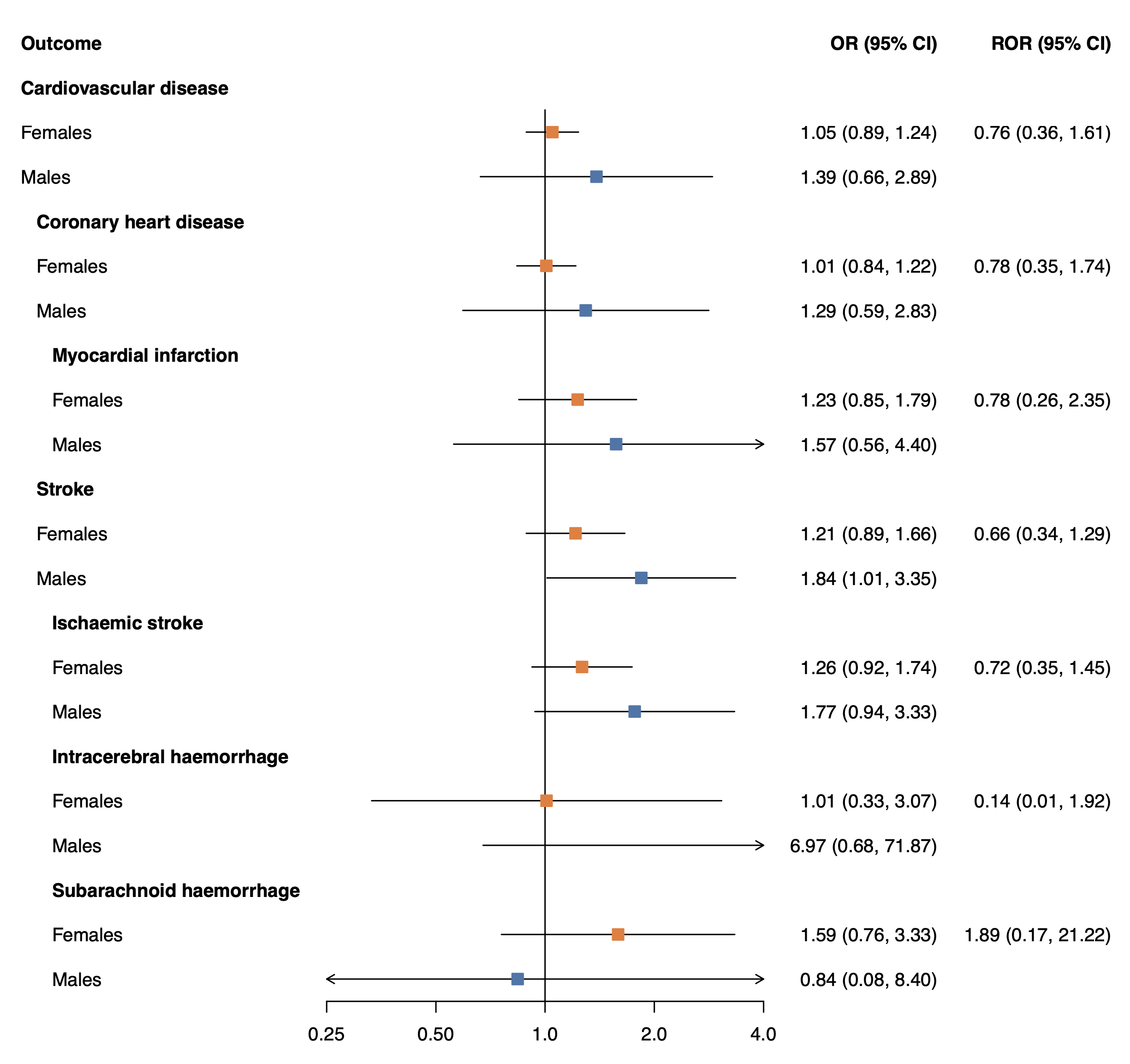
Estimates are from inverse-variance weighted MR. The odds ratios (ORs) can be interpreted as the effect per one log unit increase in genetically predicted fasting insulin level. MR analyses were performed in 320,443 UK Biobank participants. RORs present the female-to-male ratios of ORs as obtained from two separate MR analyses.
Abbreviations: CI, confidence interval; OR, odds ratio; ROR, ratio of odds ratios.
